# The role of DNA methylation in directing the functional organization of the cancer epigenome

**DOI:** 10.1101/gr.183368.114

**Published:** 2015-04

**Authors:** Fides D. Lay, Yaping Liu, Theresa K. Kelly, Heather Witt, Peggy J. Farnham, Peter A. Jones, Benjamin P. Berman

**Affiliations:** 1 Department of Biochemistry and Molecular Biology, Norris Comprehensive Cancer Center, Keck School of Medicine, University of Southern California, Los Angeles, California 90033, USA;; 2 Program in Genetic, Molecular and Cellular Biology, Keck School of Medicine, University of Southern California, Los Angeles, California 90033, USA;; 3 USC Epigenome Center, University of Southern California, Los Angeles, California 90033, USA;; 4 Van Andel Institute, Grand Rapids, Michigan 49503, USA;; 5 Department of Preventive Medicine, University of Southern California, Los Angeles, California 90033, USA

## Abstract

The holistic role of DNA methylation in the organization of the cancer epigenome is not well understood. Here we perform a comprehensive, high-resolution analysis of chromatin structure to compare the landscapes of HCT116 colon cancer cells and a DNA methylation-deficient derivative. The NOMe-seq accessibility assay unexpectedly revealed symmetrical and transcription-independent nucleosomal phasing across active, poised, and inactive genomic elements. DNA methylation abolished this phasing primarily at enhancers and CpG island (CGI) promoters, with little effect on insulators and non-CGI promoters. Abolishment of DNA methylation led to the context-specific reestablishment of the poised and active states of normal colon cells, which were marked in methylation-deficient cells by distinct H3K27 modifications and the presence of either well-phased nucleosomes or nucleosome-depleted regions, respectively. At higher-order genomic scales, we found that long, H3K9me3-marked domains had lower accessibility, consistent with a more compact chromatin structure. Taken together, our results demonstrate the nuanced and context-dependent role of DNA methylation in the functional, multiscale organization of cancer epigenomes.

Eukaryotic genomes are controlled by interrelated and mitotically heritable sets of epigenetic mechanisms, consisting of DNA methylation, nucleosome positioning, and histone modifications, which cooperate to determine gene activation potential. DNA methylation, the most clinically relevant epigenetic feature, is a covalent addition of a methyl group on the cytosine of CpG dinucleotides. In mammals, DNA methylation is required for the suppression of transcriptional activity in normal cells, particularly during imprinting, X-inactivation, and silencing of retrotransposons. Recent studies have suggested that DNA methylation may play a role in fine-tuning or reinforcing gene silencing rather than initiating it (Jones 2012; Rivera and Ren 2013). The majority of the approximately 28 million CpG sites in the human genome are normally methylated, the exception being those located in promoters, enhancers, and insulators, which can be demethylated in cell type-specific patterns (Lister et al. 2009; Cohen et al. 2011; Stadler et al. 2011; Ziller et al. 2013). Methylation patterns are faithfully copied in a cell cycle-dependent process mediated by DNA methyltransferases DNMT1 and DNMT3A/B, which preferentially bind to nucleosomes (Jones and Liang 2009; Sharma et al. 2011).

The nucleosome is the primary unit of chromatin structure and consists of 147 bp of DNA wrapped around a histone octamer of H2A/B, H3, and H4. The organization of nucleosomes, along with covalent modifications on the histone tails, are important for maintaining a balance between compaction and accessibility of the genome by transcription factors and other DNA binding proteins during cellular processes such as transcription, replication, and repair (Li et al. 2007; Cairns 2009). The precise positioning of nucleosomes at gene promoters as well as noncoding regulatory elements is an evolutionarily conserved mechanism that plays a major role in eukaryotic transcriptional regulation (Lorch et al. 1987; Schones et al. 2008; Kelly et al. 2010; Bell et al. 2011). Various factors such as underlying DNA sequences, sequence-specific DNA binding factors, and ATP-dependent nucleosome remodelers are involved in the positioning of nucleosomes (Tillo and Hughes 2009; Tillo et al. 2010; Bell et al. 2011; Valouev et al. 2011; Zhang et al. 2011). The role of DNA methylation in directing nucleosome positioning in mammals is poorly defined and highly controversial (Chodavarapu et al. 2010; Bell et al. 2011; Valouev et al. 2011; Portela et al. 2013).

Epigenetic changes, in particular the aberrant DNA methylation and silencing of CpG island (CGI) promoters, are a common signature of cancer (Baylin and Jones 2011). This early observation, along with the fact that >60% of promoters are located within CGIs, has driven the focus on CGIs as a model of study for epigenetic regulation (Tazi and Bird 1990; Irizarry et al. 2009; Deaton and Bird 2011; Portela et al. 2013). Unbiased whole-genome methylation platforms, however, have revealed that methylation changes at other regulatory regions such as CGI shores, non-CGI promoters, and enhancers may also play a role in tumorigenesis (Doi et al. 2009; Irizarry et al. 2009; Rach et al. 2011; Berman et al. 2012; Aran and Hellman 2013; Hovestadt et al. 2014; Taberlay et al. 2014). Epigenomic mapping projects such as the NIH Roadmap Epigenomics Program and the ENCODE Project Consortium (Bernstein et al. 2010; The ENCODE Project Consortium 2012) have shown that distinct epigenetic marks are highly correlated and form consistent epigenetic states (Rivera and Ren 2013). Histone marks and variants have been broadly categorized as active (H3K27ac), permissive (H3K4me1-3, H2A.Z), or repressive (H3K27me3, H3K9me3); and computational methods such as chromHMM have been used to define distinct combinatorial states such as active versus poised promoters and enhancers (Creyghton et al. 2010; Ernst et al. 2011; Rada-Iglesias et al. 2011). Despite extensive study on DNA methylation changes in cancer, we still lack an understanding of how DNA methylation changes alter these integrated chromatin states (Jones 2012).

Here, we compare a colon cancer cell line HCT116 with its almost completely unmethylated derivative, DKO1, to evaluate the effects of DNA methylation on nucleosome positioning and histone modifications. Our study couples NOMe-seq with histone ChIP-seq and RNA-seq to generate integrated maps of chromatin architecture and gene expression. Using the DKO1 model, which was genetically engineered to have a complete depletion of DNMT3B and hypomorphic expression of DNMT1 (Rhee et al. 2002), we profile the focal and long-range changes in chromatin structures and elucidate how perturbations in global DNA methylation pattern may directly alter the functional organization of the cancer epigenome and thereby, gene transcription (Rhee et al. 2002; Egger et al. 2006; Sharma et al. 2011; De Carvalho et al. 2012).

## Results

### NOMe-seq detects nucleosome depletion at a subset of genomic enhancers in methyltransferase-deficient DKO1 cells

**Figure 1.**
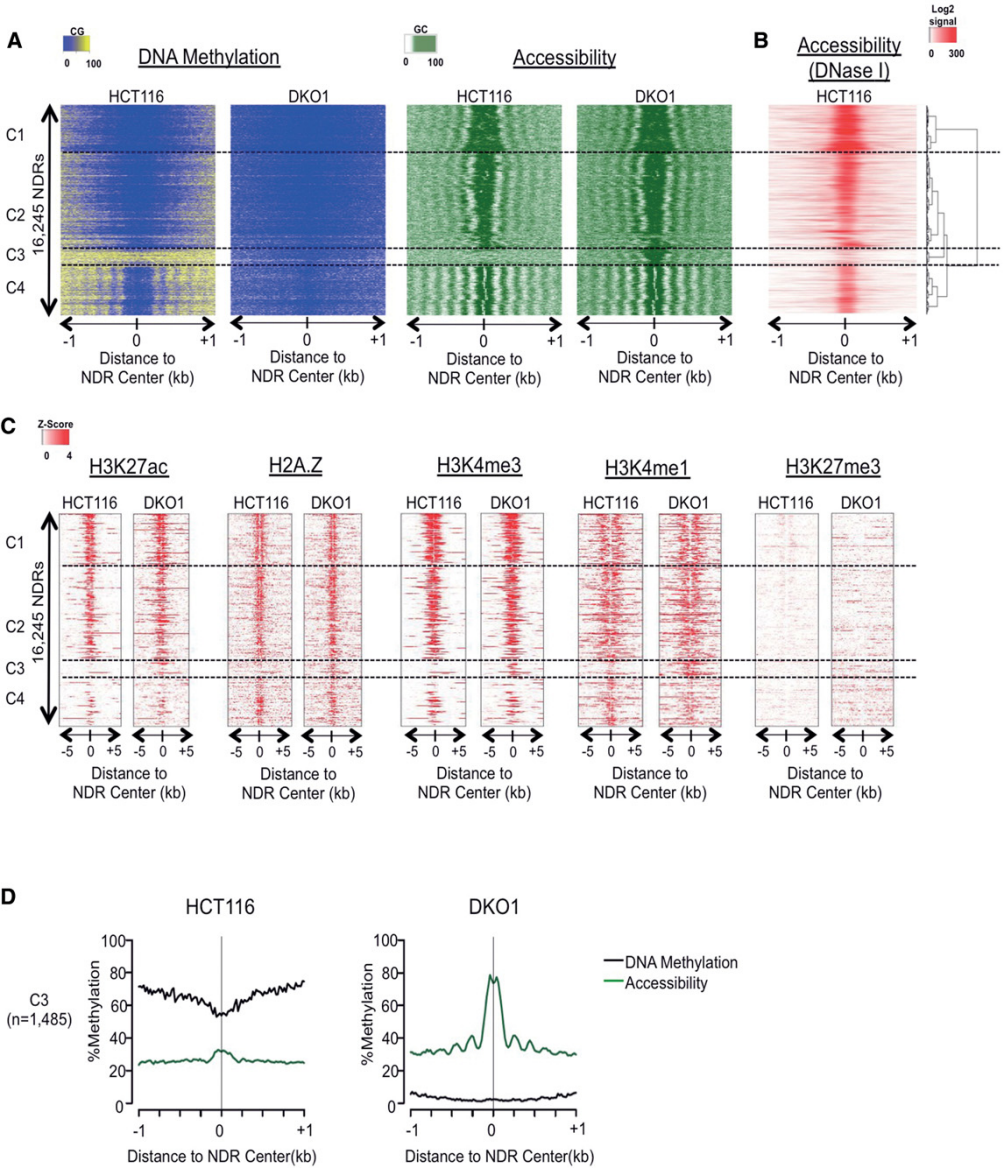
NOMe-seq detects nucleosome depletion at a subset of genomic enhancers in methyltransferase-deficient DKO1 cells. (*A*) Our hidden Markov model (HMM) identified all methyltransferase accessible regions (MARs), and those longer than 100 bp were considered nucleosome-depleted regions (NDRs). The heatmap includes all NDRs that were overlapping between the two biological replicates of either HCT116 and DKO1 cells (*n* = 16,245). NOMe-seq methylation levels (CG methylation for “DNA Methylation” and GC methylation for “Accessibility”) were aligned based on the center of the NDR and extended by ±1 kb. NDRs were hierarchically clustered based on the accessibility within ±250 bp of NDR centers in both cell types, and dashed horizontal lines separate the four top-level clusters. (*B*) ENCODE DNase hypersensitivity signal intensity is plotted for HCT116 cells for the same genomic regions in *A*. (*C*) Enrichment level for each histone mark was calculated as *Z*-score against the genomic background, and the value was plotted ±5 kb from the center of the NDRs for the same regions in *A*. (*D*) Average methylation (black) and accessibility (green) levels are shown for the altered cluster C3 (plots for the invariant clusters C1, C2, and C4 are shown in Supplemental Fig. 1D.)

Accessible or nucleosome-depleted regions (NDRs) are a distinct feature of active regulatory elements (Kelly et al. 2012; Rivera and Ren 2013). Using NOMe-seq, we characterized the relationship between DNA methylation and chromatin accessibility by analyzing two biological replicates each of HCT116 and the severely hypomethylated DKO1 cells (Supplemental Table 1). We developed a hidden Markov model (HMM) approach to identify 16,245 NDRs present in one or both cell types (Fig. 1A) and confirmed the accessibility of the HCT116 NDRs by examining the publicly available ENCODE DNase hypersensitivity mapping data for HCT116 (Fig. 1B). We hierarchically clustered the NDRs into four distinct clusters (C1–C4) and found that the vast majority (those within clusters C1, C2, and C4) were conserved between cell types, and the results were consistent in the second NOMe-seq replicate (Supplemental Fig. 1A–C). Most of the NDRs in clusters C1 and C2 were flanked on both sides by strongly phased nucleosomes (i.e., “symmetrical” phasing) (Fig. 1A; Supplemental Fig. 1D). The C1 and C2 NDRs were also associated with strong enrichment of the active H3K27ac and permissive H2A.Z, H3K4me3, and H3K4me1 marks (Fig. 1B) and overlapped active and weakly active CGI promoters in both cell types (Supplemental Fig. 1E). NDRs in cluster C4 consisted of weak enhancers or insulators characterized by CTCF binding sites (Supplemental Fig. 1E). The highly consistent phasing patterns around the C4 CTCF sites (Supplemental Fig. 1D) substantiated our earlier findings that DNA methylation rates are significantly higher within internucleosome linker regions than within nucleosome cores (Kelly et al. 2012; Berman et al. 2013; Taberlay et al. 2014). DNA methylation loss in DKO1 cells did not affect the strong nucleosome positioning around CTCF, further demonstrating that the effects of DNA methylation on nucleosome organization are limited and context specific.

The C3 cluster contained 1485 NDRs that were specific to DKO1 cells in both replicates (Fig. 1A; Supplemental Fig. 1A). In HCT116, the C3 regions were characterized by high DNA methylation and low chromatin accessibility. In addition to NDRs, these regions acquired phasing of surrounding nucleosomes in DKO1 (Supplemental Fig. 1D). These regions gained permissive and active histone marks in DKO1 that were mostly absent in HCT116 cells and were highly enriched for the “strong enhancer” chromHMM state in DKO1 cells (Supplemental Fig. 1E). Interestingly, many of these regions were premarked with low-level H3K4me1 in HCT116, reminiscent of the H3K4me1 premarking of “poised” enhancers and promoters in development (Creyghton et al. 2010; Rada-Iglesias et al. 2011). Furthermore, C3 NDRs were significantly enriched for specific transcription factor binding motifs ARNT (also known as HIF-1beta) and FOS (AP-1 motif for FOS/JUN dimer, Supplemental Fig. 1F), demonstrating the highly specific nature of these enhancer changes. As a further indication of specificity, enhancer regions defined by ENCODE chromHMM data and NOMe-seq that were active in an unrelated cell type (leukemia-derived K562 cells), remained inactive and did not gain NDRs or nucleosomal phasing in DKO1 cells (Supplemental Fig. 1G).

### Loss of DNA methylation results in the reorganization of nucleosomes and the acquisition of active and poised chromatin marks at CGI promoters

**Figure 2.**
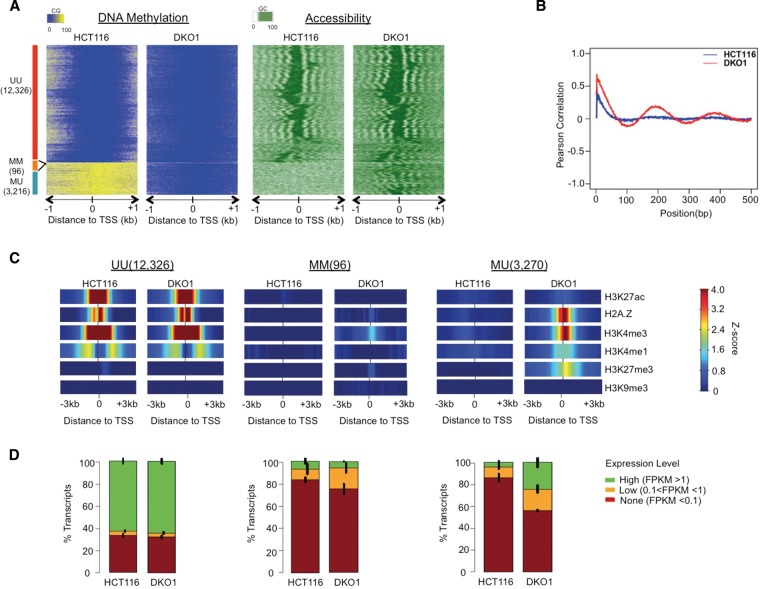
Loss of DNA methylation results in the reorganization of nucleosomes and the acquisition of active and poised chromatin marks at CGI promoters. (*A*) Average NOMe-seq methylation levels (CG methylation for “DNA Methylation” and GC methylation for “Accessibility”) were aligned to transcription start sites (TSSs) of 15,638 CGI promoters and extended by ±1 kb. The heatmap was organized into four sections based on the DNA methylation levels: Unmethylated in HCT116 and Unmethylated in DKO1 (UU); Methylated in HCT116 and Methylated in DKO1 (MM); and Methylated in HCT116 and Unmethylated in DKO1 (MU). Within each class, promoters were ordered based on hierarchical clustering of the accessibility pattern in DKO1 cells (a similar clustering based on the accessibility of HCT116 cells is shown in Supplemental Fig. 2A). (*B*) In each cell type, Pearson correlations were calculated based on methylation levels between pairs of GCs at each possible distance from one another. Only those pairs in which both GCs were from 0 to 700 bp downstream from the TSS were considered. (*C*) Within each promoter class, the *Z*-score enrichment level of each histone mark was extended to ±3 kb around the TSS and averaged (see Methods for *Z*-score enrichment definition). (*D*) FPKM transcript values for all genes were divided into three levels, and the fraction within each level is shown along with error bars indicating the standard error across two RNA-seq biological replicates.

In the clustering described above, we observed a consistent gain of the poised CGI promoter chromHMM state across the two largest clusters, C1 and C2 (Supplemental Fig. 1E). To systematically address the effects of DNA methylation loss on CGI promoters, we stratified all CGI promoters based on their methylation status in the two cell types: UU (unmethylated in both HCT116 and DKO1), MU (unmethylated only in DKO1), and MM (methylated in both). We investigated chromatin features of the different CGI promoter classes by clustering all promoters within the class based on DKO1 accessibility (Fig. 2A). The majority of CGI promoters (12,326) fell into the UU class, consistent with the idea that CGIs are generally devoid of DNA methylation, even in cancer (Weber et al. 2007; Gebhard et al. 2010; Deaton and Bird 2011). UU promoters had an open architecture, with a highly accessible NDR region flanked by at least three well-phased nucleosomes in both the 5′ and 3′ directions from the TSS (Fig. 2A), and were consistent in both NOMe-seq replicates (Supplemental Fig. 2). The remainder of CGI promoters (3312) were methylated in HCT116 cells. A small set of these were in the MM class (96) and retained a closed chromatin architecture in DKO1 cells; these may represent a small but important set of genes with selective pressure to retain an inactive chromatin configuration (De Carvalho et al. 2012). The remaining 3216 CGI promoters methylated in HCT116 fell into the MU class. These showed a dramatic reorganization of the surrounding chromatin in DKO1 cells, with newly positioned nucleosomes in both the 5′ and 3′ directions (Fig. 2A). The de novo phasing around MU promoters was not an artifact of our heatmap clustering method (Supplemental Fig. 2A); to quantify the increased nucleosomal phasing in DKO1 cells, we calculated the positional autocorrelation of accessibility levels for all MU regions (Fig. 2B).

To understand the functional relevance of the striking nucleosome organization changes, we analyzed histone modifications and gene expression patterns in HCT116 and DKO1 cells. Histone modifications at UU promoters remained consistent between HCT116 and DKO1 cells, including the presence of the histone variant H2A.Z, the active mark H3K27ac, and the permissive marks H3K4me3 and H3K4me1 (Fig. 2C, Supplemental Fig. 3A). ChromHMM states and expression levels were largely unchanged, with most promoters having high expression and active promoter status (Fig. 2D; Supplemental Fig. 3B). In contrast, CGI promoters that were methylated in HCT116 (the MM and MU classes) were largely devoid of all active, permissive, and repressive histone marks in HCT116. In HCT116 cells, these promoters were unexpressed (Fig. 2D) and associated with inactive promoter states (Supplemental Fig. 3B). Those that retained residual DNA methylation in DKO1 cells (the MM class) had relatively few changes in histone modifications (Fig. 2C), gene expression (Fig. 2D), or chromatin state (Supplemental Fig. 3B).

**Figure 3.**
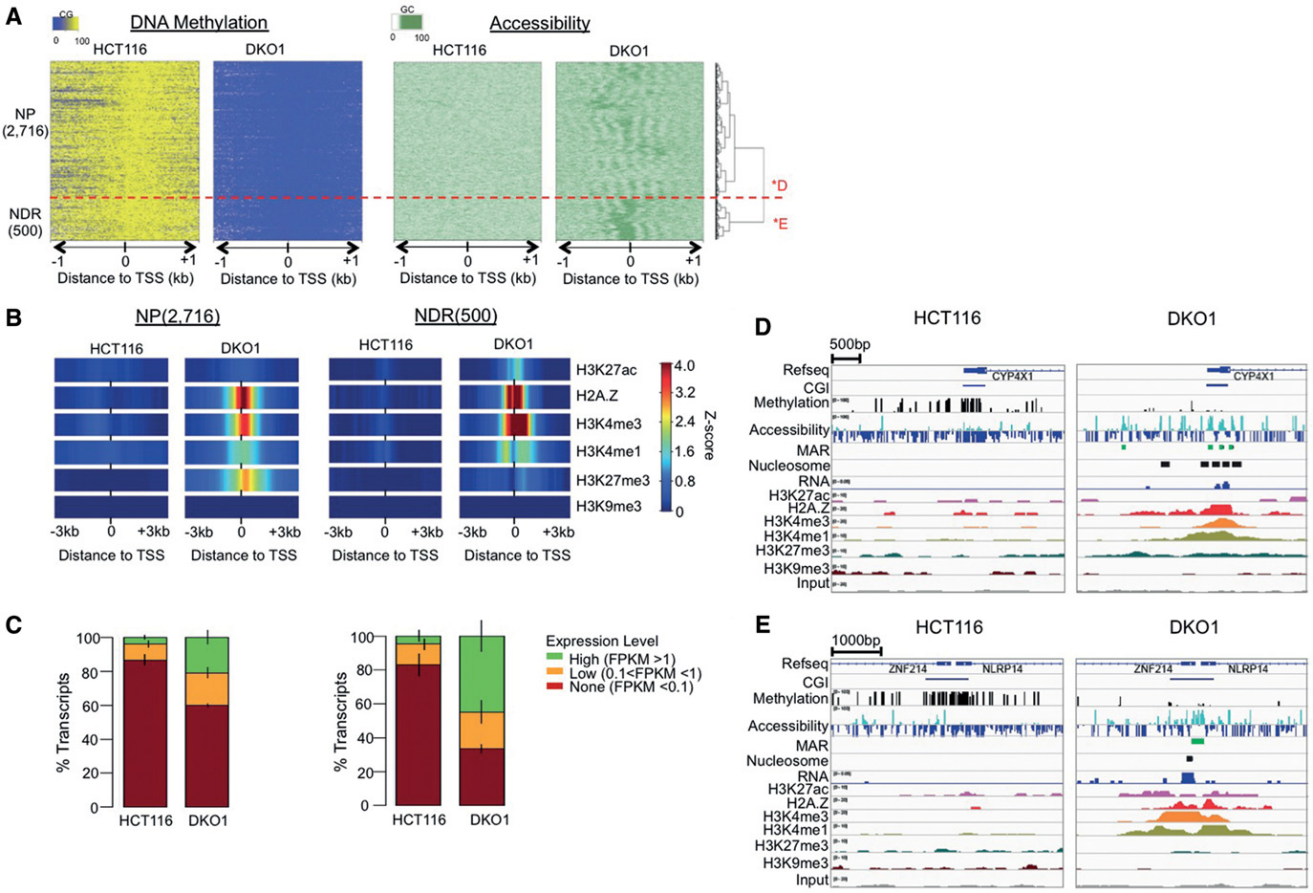
CGI promoters losing methylation acquire either an active or poised chromatin state, each with a distinct nucleosomal profile and histone modifications that resemble their respective patterns in normal colon cells. (*A*) NOMe-seq reads were aligned to 3216 MU CGI TSS from Figure 2 and hierarchically clustered based on the accessibility level in DKO1 cells. Two top-level clusters were detected in the unsupervised clustering: NP gained nucleosome positioning alone, while NDR promoters also gained nucleosome depletion. (*B*) Enrichment of each histone mark for the two clusters, displayed as in Figure 2. (*C*) Transcript level for each promoter class as displayed in Figure 2. (*D*) IGV browser view of NP gene *CYP4X1* and (*E*) NDR gene *ZNF214* (labeled as *D and *E in *A*). IGV views show DNA methylation (CG) and accessibility (GC) NOMe-seq methylation levels and ChIP-seq histone marks, with HCT116 and DKO1 plotted side by side. Methyltransferase accessible regions (MARs) and mononucleosomes were identified by our hidden Markov model (HMM), as described in Methods.

In contrast to MM promoters, those that completely lost methylation in DKO1 (the MU class) gained the permissive H2A.Z, H3K4me3, and H3K4me1 marks as well as the repressive H3K27me3 mark (Fig. 2C). The acquisition of H3K27me3 with the abolishment of DNA methylation, but not the other repressive mark H3K9me3, was consistent with earlier reports (Jin et al. 2009; Komashko and Farnham 2010). This is particularly intriguing due to the association of many of these cancer-hypermethylated CGI promoters with H3K27me3-containing poised or bivalent states in normal cell types (Ohm et al. 2007; Schlesinger et al. 2007; Widschwendter et al. 2007). Furthermore, at MU promoters, H3K4me1 was localized directly over the TSS, in contrast to its distribution at active UU promoters where two peaks flank a central NDR (Fig. 2C; Supplemental Fig. 3A). This central H3K4me1 pattern was consistent with the H3K27me3-containing poised promoter state described in earlier studies (McGarvey et al. 2008; Hawkins et al. 2010).

### CGI promoters losing methylation acquire either an active or poised chromatin state, each with a distinct nucleosomal profile, and histone modifications that resemble their respective patterns in normal colon cells

When investigated in detail, the 3216 MU promoters fell into two distinct subclusters, one lacking an NDR but gaining nucleosome phasing (2716 “NP” promoters) and a second gaining phasing that flanked an NDR (500 “NDR” promoters) (Fig. 3A). The NP class had strong enrichment of the poised H3K27me3 mark, in contrast to the enrichment of the active H3K27ac in the NDR class (Fig. 3B), which was consistent across replicates (Supplemental Fig. 4A). This suggested a novel concept that highly organized nucleosomes are compatible with promoters in the Polycomb/poised state. Forty-five percent of the NDR genes became highly expressed in DKO1 cells, compared to 21% of NP genes (Fig. 3C). This result is consistent with previous observations that gene reactivation following DNA demethylation requires the formation of accessible or NDR regions at the TSS (Lin et al. 2007; Yang et al. 2012). The acquisition of H3K27me3 and phasing without nucleosome depletion can be seen at the NP gene *CYP4X1* gene promoter (Fig. 3D), in contrast to an NDR gene *ZNF214* (Fig. 3E).

**Figure 4.**
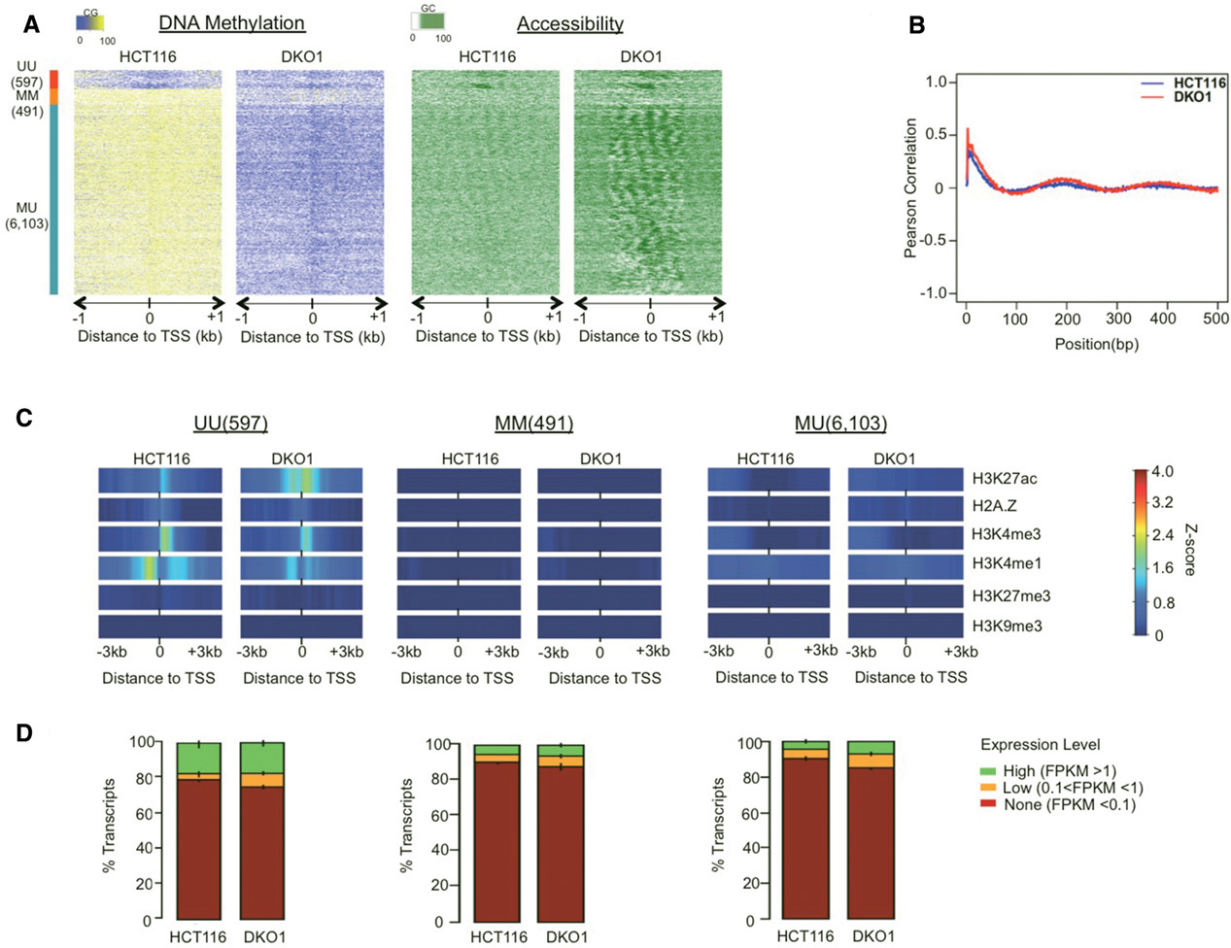
The loss of DNA methylation has little effect on the chromatin structure of non-CGI promoters. (*A*) NOMe-seq methylation levels were aligned to 7191 TSSs for annotated non-CGI promoters and displayed as in Figure 2. (*B*) Pearson autocorrelation of GC (accessibility) methylation levels was calculated independently for each cell type and displayed as in Figure 2. (*C*) Within each promoter class, the *Z*-score enrichment level of each histone mark is displayed as in Figure 2. (*D*) FPKM transcript values for all genes were divided into three levels and displayed as in Figure 2.

The gain of the Polycomb/poised signature at NP promoters prompted us to investigate the status of these regions in normal colon profiles from the Roadmap Epigenomics Consortium (Bernstein et al. 2010), since the presence of the Polycomb mark in normal precursor cells is a strong predictor of methylation gain in cancer (Ohm et al. 2007; Schlesinger et al. 2007; Widschwendter et al. 2007) and is postulated to be part of a global “epigenetic switch” from Polycomb repression to DNA methylation (Gal-Yam et al. 2008; Jin et al. 2009). Indeed, the histone marks and expression levels of NP genes in DKO1 cells strongly resembled those of normal colonic mucosa (Supplemental Fig. 5, left), suggesting that the Polycomb to DNA methylation epigenetic switch in cancer may be reversed by the removal of DNA methylation (McGarvey et al. 2008). Remarkably, the active histone and expression patterns of NDR genes in DKO1 were also recapitulated in the patterns of normal colonic mucosa (Supplemental Fig. 5, right). The NDR promoters, unlike the NP promoters, were enriched for binding motifs for specific transcription factors such as SP1 and NRF1 (Supplemental Fig. 6), typical “housekeeping” transcription factors that normally protect CGI promoters from methylation in both normal and cancer cells (Gebhard et al. 2010; Berman et al. 2012). How a small number of normally active promoters like these can gain methylation in cancer (along with a larger set of poised promoters which may be explained by the epigenetic switch) is an open question, but our results here suggest that many could be reversed by the removal of DNA methylation. Understanding this process may lead to insights into other similarly silenced genes like *MLH1* in colorectal cancer and *BRCA1* in breast and ovarian cancer.

**Figure 5.**
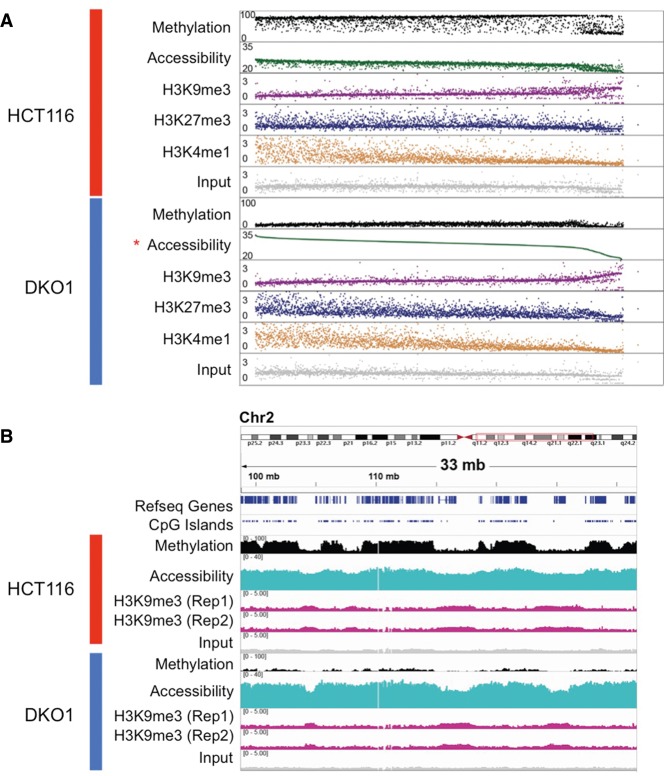
Long H3K9me3-marked heterochromatin domains have lower chromatin accessibility and partially methylated DNA. (*A*) NOMe-seq methylation levels and ChIP-seq enrichment *Z*-scores (excluding those within CpG islands) were averaged within each 1-Mb window in the genome. Each dot in each track shows the average value for the same 1-Mb window and which are ranked based on accessibility level in DKO1 (starred). (*B*) IGV browser view for a 33-Mb genomic window on Chr 2q. Multiple biological replicates are shown for H3K9me3 to illustrate the reproducibility in H3K9me3 domain shrinking.

### The loss of DNA methylation has little effect on the chromatin structure of non-CGI promoters

Non-CGI promoters have regulatory characteristics distinct from CGI promoters. As a group, they are more likely to be expressed in a tissue-specific pattern, be associated with TATA boxes, and initiate transcription from a particular base pair rather than many possible initiation sites within a “broad” region (Rach et al. 2011); they are also almost never repressed by Polycomb-associated H3K27me3 (Mohn et al. 2008). By analyzing non-CGI using the same methods as CGI promoters (Fig. 4), we found that the two promoter types differed dramatically in their response to DNA methylation loss. Unlike CGI promoters, most non-CGI promoters are methylated, so the overwhelming majority (85%) fell into the MU state. Only a small subset of non-CGI promoters had NDRs, and these were limited to the UU class (Fig. 4A; Supplemental Fig. 7). Unlike CGI promoters, we detected weakly organized arrays of nucleosomes at most MU promoters in HCT116 cells (Fig. 4A). Well-positioned nucleosomes have often been described as a feature of unmethylated and permissive promoters, but our finding suggests that nucleosome organization can occur at non-CGI promoters and is independent of methylation status. Autocorrelation analysis showed that the extent of phasing changed little between HCT116 and DKO1 cells (Fig. 4B). The loss of DNA methylation in DKO1 cells also did not appear to influence the histone modification patterns of non-CGI MU promoters, which were devoid of both permissive and repressive histone marks (Fig. 4C; Supplemental Fig. 8A,B) and were largely inactive (Fig. 4D). These observations suggested that the loss of DNA methylation alone was insufficient to remodel and reactivate a major class of non-CGI promoters as it does for CGI promoters, and DNA methylation itself may not play an important role in modulating the chromatin landscape of non-CGI promoters in cancer.

### Long H3K9me3-marked heterochromatin domains have lower chromatin accessibility and partially methylated DNA

Having established the effects of genome-wide loss of DNA methylation on the focal chromatin structure of enhancers and promoters, we next investigated the effects on higher-order chromatin domains (Lister et al. 2011; Berman et al. 2012; Hon et al. 2012; Bert et al. 2013). We ranked all nonoverlapping 1-Mb windows in the genome based on the average GpC accessibility signal in DKO1 cells (Fig. 5A), which was largely concordant between HCT116 and DKO1 at this domain scale. We observed a distinct set of windows at the right end of the Figure 5A plot that had relatively low accessibility and DNA hypomethylation in both HCT116 and DKO1. These regions of lower accessibility coincided with partially methylated domains or PMDs (Fig. 5B), known to coincide with heterochromatic late-replicating regions in cancer (Hansen et al. 2011; Lister et al. 2011; Berman et al. 2012; Hon et al. 2012; Hovestadt et al. 2014). The low accessibility observed within these domains (Fig. 5A,B) may be due to a compact chromatin structure (see Discussion) and coincided with the H3K9me3 mark observed in both cell types (Fig. 5A,B; Supplemental Fig. 9A).

Consistent with earlier reports (Hon et al. 2012; Gifford et al. 2013), the H3K9me3 domains were mutually exclusive with H3K27me3 and H3K4me1. As illustrated by a representative region on Chromosome 2 (Fig. 5B), many of the H3K9me3 domains became shorter in DKO1 cells than in the HCT116 cells, perhaps due to reestablishment of H3K27me3 near the domain boundaries occurring as part of the epigenetic switch (see Discussion). Surprisingly, the H3K9me3 domains overlapped domains of H3K4me3 (Supplemental Fig. 9A,B). Large H3K4me3 domains have previously been associated with the transcriptional consistency of lineage-specific regions and activation of the cancer genome (Bert et al. 2013; Benayoun et al. 2014). The coexistence of H3K4me and H3K9me3, however, has not been described, and its significance will require further investigation.

## Discussion

The role of epigenetics in transcriptional regulation is key to understanding the establishment of normal mammalian phenotypes and common diseases (Baylin and Jones 2011). The remarkable growth in the field of integrative epigenomics has revealed that the functions of DNA methylation may be more nuanced than previously understood and act together with other chromatin features as components of integrated epigenetic states (Jones 2012; Bergman and Cedar 2013; Rivera and Ren 2013). Although DNA methylation changes have been associated with changes in transcriptional state, our understanding of how DNA methylation influences nucleosomal changes has remained inconclusive (Chodavarapu et al. 2010; Valouev et al. 2011; Kelly et al. 2012; Berman et al. 2013). Understanding the specific role that DNA methylation plays in gene regulation thus requires a holistic examination of its interactions with other epigenetic mechanisms, including histone modifications and nucleosome positioning.

Here, we have investigated the epigenetic changes associated with the abolishment of DNA methylation in cancer cells, combining NOMe-seq for single base-pair resolution maps of DNA methylation and nucleosome positioning with ChIP-seq for mapping modified histones. At some genomic elements, such as CTCF insulators and non-CpG island (non-CGI) promoters, we found little change between the colon cancer cells (HCT116) and the methylation-deficient derivative cell line (DKO1). For enhancers, we found that a small but significant subset was affected by loss of DNA methylation, with coordinated changes in nucleosome positioning and histone modifications. Loss of methylation only affected a small subset of potential enhancers, with sequence motifs suggesting that the specificity involves transcription factors such as ARNT and FOS (AP-1 motif for FOS/JUN dimer). These changes could be a direct (i.e., *cis-*acting) result of methylation loss in a model where transcription factor complexes gain increased access to DNA with the loss of methylation. However, such a model is not required to explain these changes, as transcription factor binding alone is sufficient to demethylate fully methylated enhancer sequences in vivo, and demethylation occurs genome-wide in vivo in short (several hundred bp) regions that are defined by transcription factor binding sites (Stadler et al. 2011; Berman et al. 2012; Benveniste et al. 2014). The simpler and perhaps more likely model is that the epigenomic changes at DKO1 enhancers are indirect (*trans-*acting), with global DNA methylation loss activating functional pathways that lead to changes in transcription factor activity. Such indirect changes are not unexpected, as DKO1 cells adapted to the loss of DNA methylation over the course of many generations.

Our results at methylated CpG island promoters suggest a more global and therefore more likely *cis* mechanism. CGI promoters that were methylated in HCT116 cells (unlike non-CGI promoters, discussed below) responded to loss of DNA methylation with increased nucleosomal phasing and an acquisition of either active or repressive histone marks. One subset of these promoters acquired nucleosome-depleted regions along with active histone marks (NDR subset), whereas a much larger subset gained nucleosome phasing but not nucleosome depletion, along with Polycomb-repressive marks characteristic of a poised chromatin state (NP subset). Strikingly, the histone modification patterns acquired in these two subsets recapitulated their respective patterns in normal colonic mucosa. This suggests that DNA methylation has the ability to suppress multiple classes of CGI promoters during tumorigenesis, both active and poised, and that this suppression may be reversed in a context-specific manner when DNA methylation is removed. The NP class seems likely to represent a direct, *cis*-acting consequence of DNA methylation loss. First, it is relatively nonspecific, affecting 85% of CGI promoters that become demethylated in HCT116. Second, this class contains the majority of CGI promoters associated with the Polycomb (poised) state in normal colonic mucosa, methylated en masse in cancers via a global “epigenetic switch” (Gal-Yam et al. 2008; Baylin and Jones 2011). The fact that these promoters regain their Polycomb-associated marks in DKO1 cells suggests that Polycomb activity is directly repressed by DNA methylation in HCT116 cells and can be reestablished when DNA methylation is removed. The situation is somewhat less clear at the 15% of methylated CGI promoters that regain active status in DKO1 cells. Although they do reestablish a normal colon-like chromatin state, they represent a tiny fraction of all CGI promoters active in normal colon. How these promoters become specifically methylated in cancer is unknown, and our transcription factor motif analysis here uncovers no additional candidates—the transcription factor binding motifs enriched in NDR promoters in Supplemental Figure 6 belong to the same general transcription factors found at all normally active CpG islands (Gebhard et al. 2010; Berman et al. 2012). This was in contrast to the motifs we identified at remodeled enhancers, which belonged to cancer-related transcription factors. This makes it difficult to conclude whether NDR-acquiring CGI promoters are more likely to be the result of a *cis* versus *trans* mechanism, but this question is central to understanding how CGI promoters for cancer driver genes like *MLH1*, *BRCA1*, and *VHL* become epigenetically silenced in cancer.

Our results here underscore the fundamental differences between CpG island (CGI) and non-CGI promoters in mammals. In HCT116 cells, methylated non-CGI promoters tended to have weak nucleosome phasing without nucleosome depletion; in contrast, methylated CGI promoters had no detectable phasing. The presence of weakly phased nucleosomes at methylated non-CGI promoters had not been detected previously, even in our earlier NOMe-seq study (Kelly et al. 2012). This was likely due to improvements in our NOMe-seq protocol and several-fold greater sequencing coverage, but we cannot rule out cell type differences (our earlier study focused on cultured fibroblast cells, whereas here we focus on cancer cells). Weak nucleosome phasing at non-CGI promoters is consistent with the intrinsic affinity of nucleosomes for G/C rich sequences, which are abundant at promoters due to the presence of G/C rich transcription factor binding sites (Tillo et al. 2010). It must be noted that understanding the relative phasing of nucleosomes relative to promoters is only as good as our promoter annotations. Here we have used standard annotations derived from a variety of RNA sequencing approaches; however, the availability of large promoter databases determined using 5′ Cap Analysis of Gene Expression (CAGE) mapping, will improve our ability to resolve phasing patterns relative to precise transcriptional initiation sites (FANTOM Consortium and the RIKEN PMI and CLST [DGT] et al. 2014).

Long (megabase-scale) domains of H3K9 methylation have been linked to topological domains (Dixon et al. 2012) and associated with the nuclear lamina territory (Guelen et al. 2008) and late replication timing (Hansen et al. 2010). In cancer, these domains tend to become hypomethylated (Hansen et al. 2011; Berman et al. 2012). Here, we show that NOMe-seq is able to detect these regions as domains of relatively inaccessible DNA, presumably due to the condensed structure of constitutive heterochromatin, which acts as a diffusion barrier in vivo (Bancaud et al. 2009). Domains of H3K9me3 and inaccessibility were highly overlapping and often became shorter in DKO1 cells (Fig. 5B). Long domains of H3K27me3 tend to occur just outside the edges of H3K9me3 domains (Guelen et al. 2008), perhaps mediating developmental gene silencing programs by dynamically controlling association and disassociation of genes with the nuclear lamina (Peric-Hupkes et al. 2010). We propose that the “shrinking” of H3K9me3-marked inaccessible chromatin domains that occurs in DKO1 cells is due to the reestablishment of H3K27me3 domains at the boundaries of H3K9me3, consistent with the reestablishment of H3K27me3-marked poised chromatin at CGI promoters genome-wide. This model predicts that topological domains and DNA methylation may interact to define the epigenomic landscape of cancer.

## Methods

### Cell culture

HCT116, obtained from ATCC, and DKO1 cells were cultured under recommended conditions at 37°C and 5% CO_2_ in McCoy's 5A media supplemented with 10% FBS and penicillin/streptomycin. HCT116 was obtained from ATCC, and DKO1 was a generous gift from Drs. Bert Vogelstein and Steve Baylin.

### Genome-wide nucleosome footprinting assay

NOMe-seq was performed as previously described (Kelly et al. 2012). Briefly, exponentially growing cells were washed with PBS, trypsinized, and incubated with ice-cold lysis buffer (10 mM Tris, pH 7.4, 10 mM NaCl, 3 mM MgCl_2_, 0.1 mM EDTA, and 0.5% NP-40) for 5 min on ice to isolate intact nuclei. Nuclei were washed with ice-cold wash buffer (10 mM Tris, pH7.4, 10 mM NaCl, 3 mM MgCl_2_, 0.1 mM EDTA), resuspended in ice-cold 1× GpC buffer (New England BioLabs), and treated with 200 units of M.CviPI enzyme supplemented with 1.5 μL S-adenosylmethionine (SAM) for 7.5 min with a boost of 100 units enzyme and 0.75 μL SAM for an additional 7.5 min. Genomic DNA was isolated by standard phenol-chloroform extraction and ethanol precipitation. WGBS libraries were generated using 2–5 μg of DNA as previously described and sequenced on HiSeq 2000 (Lister et al. 2009; Berman et al. 2012). Sequencing reads were mapped to the hg19 genome, and methylation levels of CpG and GpC dinucleotides were determined using the previously described pipeline (Kelly et al. 2012; Liu et al. 2012). Sequencing metrics for NOMe-seq data are listed in Supplemental Table 1.

### Hidden Markov model-based approach of NDR detection

Two-state beta-binomial HMM was adapted from a previously described method (Molaro et al. 2011) to segment regions into methyltransferase accessible regions (MARs) and methyltransferase protected regions (MPRs), based on GCH methylation in HCT116 and DKO1 cells. Training of the model was performed independently for each biological replicate. GCH methylation and read coverage were used as inputs to the Viterbi algorithm to determine the state of each individual GCH, and segments containing at least three contiguous GCHs present in the same state were required to call the MARs and MPRs. A one-tailed binomial test was used to calculate the significance level of each MAR in comparison to all MPRs present in the adjacent ±100-kb region, with only MARs having FDR-corrected *P*-value <0.01 considered significant. MARs having the length >100 bp were considered as NDRs. For the analysis of Figure 1 and Supplemental Figure 1, only NDRs that overlapped in both biological replicates in each cell line were used.

### Defining promoter methylation classes

We combined the two NOMe-seq replicates for each cell type and filtered all annotated TSSs from the UCSC knownGenes track that had at least three HCG sites and at least 10 reads from each cell type within the region from −300 to +500 bp around the TSS. We considered these TSS regions as unmethylated in a given cell type if they had an average methylation level <5%. For methylated TSSs, we used 60% and 25% as the lower cutoff for HCT116 and DKO1, respectively. These different cutoffs in the two cell types were determined based on the global distribution of methylation values in the promoter regions of each cell line. Based on this criteria, we included 15,692 CGI promoters and 7191 non-CGI promoters.

### RNA-seq

HCT116 and DKO1 cells were washed with PBS and subsequently lysed in TRIzol (Life Technologies). Total RNA from two independent cultures was purified using Direct-zol RNA MiniPrep (Zymo Research), and libraries were constructed using the poly(A) selected method of the TruSeq RNA Sample Prep Kit (Illumina) according to manufacturer's instructions. Sequencing reads were mapped to the hg19 reference genome using TopHat v.1.2 (Trapnell et al. 2009), filtering out nonuniquely mapping reads and PCR duplicates. FPKM value was calculated using Cufflinks v.2.1.1 (Trapnell et al. 2010) with the following parameters: -F 0.3 –u –b hg19.fa. Gene annotation was obtained as a GTF file from the UCSC Genome Browser (knownGene track). RNA-seq data of normal colonic mucosa was previously published and obtained from The Cancer Genome Atlas (Berman et al. 2012; The Cancer Genome Atlas Network 2012).

### ChIP-seq

ChIP assay was performed in duplicate using 50 μg of chromatin as previously described and according to ENCODE's guideline (Kelly et al. 2010; Landt et al. 2012). The following antibodies were used: H2A.Z (Abcam, ab4174); H3K4me3 (Active Motif, 39160); H3K4me1 (Active Motif, 39298); H3K27ac (Active Motif, 39297); and H3K27me3 (Active Motif, 39155). Two biological replicates of H3K9me3 and H3K36me3 as well as one biological replicate of H3K27ac and Input HCT116 ChIP-seq data were produced by the Farnham Laboratory, each as part of the ENCODE Project Consortium (The ENCODE Project Consortium 2012) and is available at (http://genome.ucsc.edu). ChIP-seq data of normal colonic mucosa was generated by the Bernstein Laboratory, as part of the Roadmap Epigenomics Mapping Consortium (Bernstein et al. 2010); all data used in this study is past the 9-mo moratorium. A complete list of sequencing metrics and GEO identifiers is included in Supplemental Table 1 for all ChIP-seq data used in this study.

Briefly, genome-wide libraries were generated from 20 ng of purified ChIP and input DNA, barcoded, and sequenced for 50 single-end reads on HiSeq 2000 using a previously described protocol (Barski et al. 2007; Kelly et al. 2012). Sequencing reads were mapped to hg19 using BWA (Li and Durbin 2009), removing nonuniquely mapping reads and PCR duplicates. All ChIP-seq reads were extended to the sequencing library's mean fragment size, which was estimated using the default setting of HOMER v.4.3's *makeTaqDirectory* command (Heinz et al. 2010). Each data set was normalized into a single value for each genomic position using the Wiggler tool with default settings and “globalmap_k20tok54” as the mappability parameter (The ENCODE Project Consortium 2012). Mean Wiggler values were calculated in 10-bp bins (Gerstein et al. 2012). To normalize variations between biological replicates, we modified a previously described method to perform *Z*-score transformation by subtracting the mean Wiggler value across the genome and dividing by the standard deviation of the genome-wide Wiggler subtraction value (Xie et al. 2013).

### ChromHMM

Segmentation and determination of chromatin states were calculated as previously described (Ernst et al. 2011). Details are further described in the Supplemental Methods. Emission and transition parameters are shown in Supplemental Figure 10.

### Source code access

All analyses were performed using the in-house Bis-tools package. All source codes are available at https://github.com/dnaase/Bis-tools and included in the Supplemental Material. Specific scripts are detailed in the Supplemental Methods.

## Data access

Data generated in this study have been submitted to the NCBI Gene Expression Omnibus (GEO; http://www.ncbi.nlm.nih.gov/geo/) under accession numbers GSE58695 and GSE64929 (NOMe-seq), GSE58638 (ChIP-seq), and GSE52429 and GSE60106 (RNA-seq).

## Competing interest statement

P.A.J. is a paid consultant for Zymo Research. T.K.K. is currently an employee of Active Motif who commercialized the NOMe-seq assay after this project was conceived. The other authors have no financial interests related to this work.

## Supplementary Material

Supplemental Material
