# Supplementary material for: The role of DNA methylation in directing the functional organization of the cancer epigenome
Source: Genome Res. 2015 Apr;25(4):467–77. doi: 10.1101/gr.183368.114 (PMC4381519; doi:10.1101/gr.183368.114)
Supplement: Supplemental Material [file supp_25_4_467__index.html]

The role of DNA methylation in directing the functional organization of the cancer epigenome — The role of DNA methylation in directing the functional organization of the cancer epigenome — Supplemental Material 

# The role of DNA methylation in directing the functional organization of the cancer epigenome

## Supplemental Material

**Files in this Data Supplement:**

- Supp Figure1.jpg
- Supp Figure2.jpg
- Supp Figure3.jpg
- Supp Figure4.jpg
- Supp Figure5.jpg
- Supp Figure6.jpg
- Supp Figure7.jpg
- Supp Figure8.jpg
- Supp Figure9.jpg
- Supp Figure10.jpg
- Supp Methods.docx
- Supp Tabl1.docx
- Supplemental source\_code.zip
