# Supplementary figures and images for: The role of DNA methylation in directing the functional organization of the cancer epigenome
Source: Genome Res. 2015 Apr;25(4):467–77. doi: 10.1101/gr.183368.114 (PMC4381519; doi:10.1101/gr.183368.114)

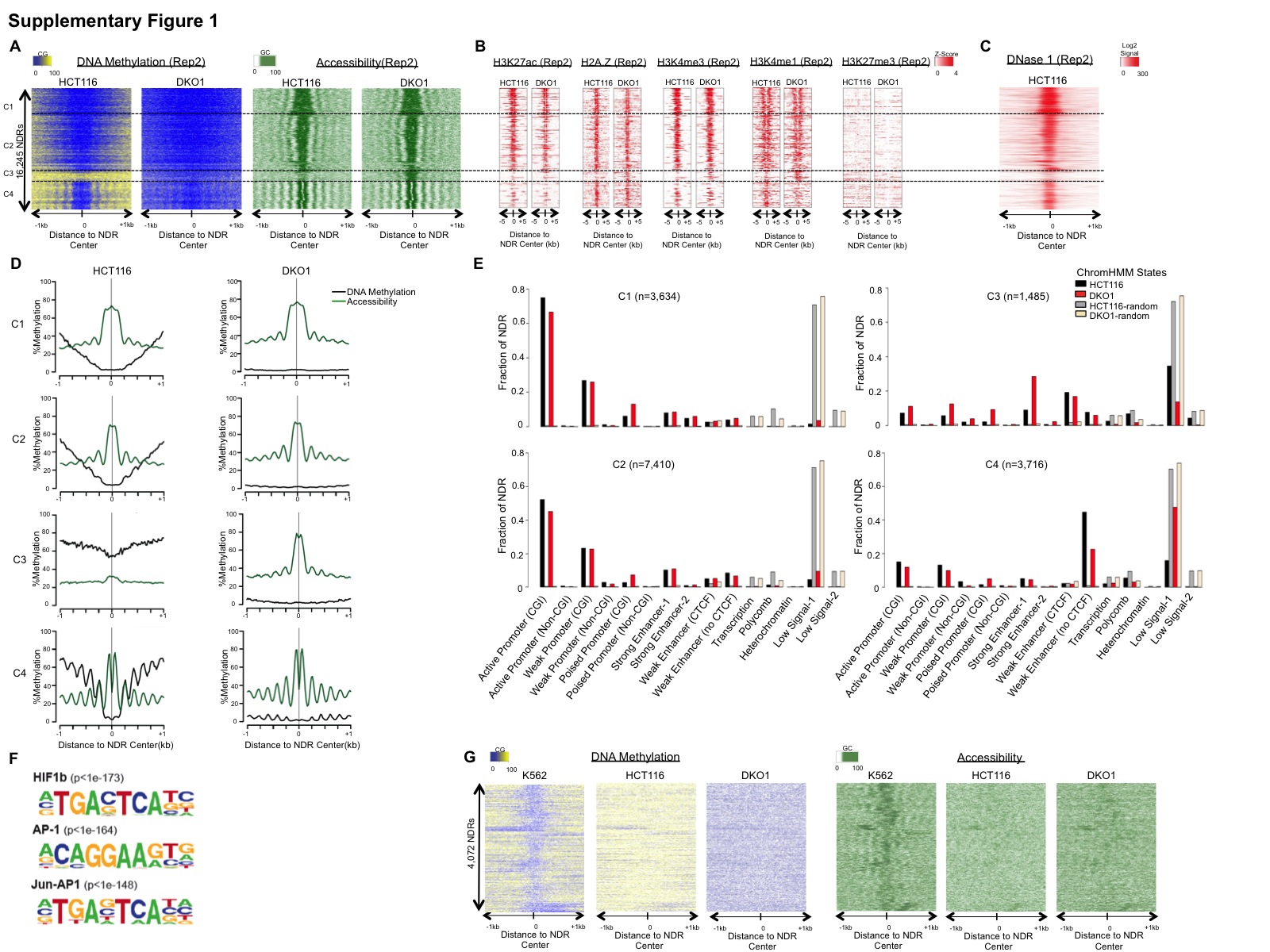

Supplement: Supplemental Material [file supp_gr.183368.114_Supp_Figure1.jpg]

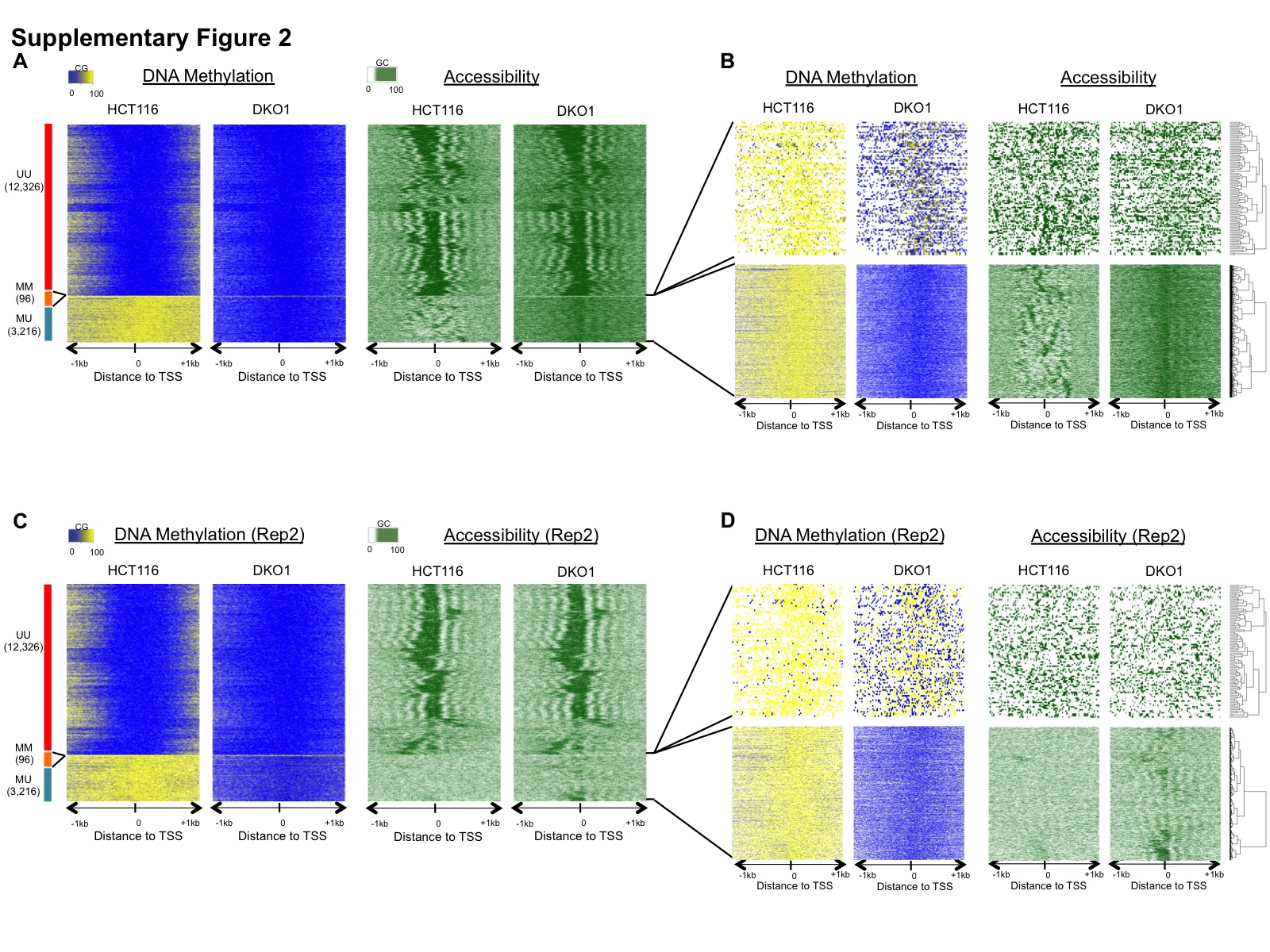

Supplement: Supplemental Material [file supp_gr.183368.114_Supp_Figure2.jpg]

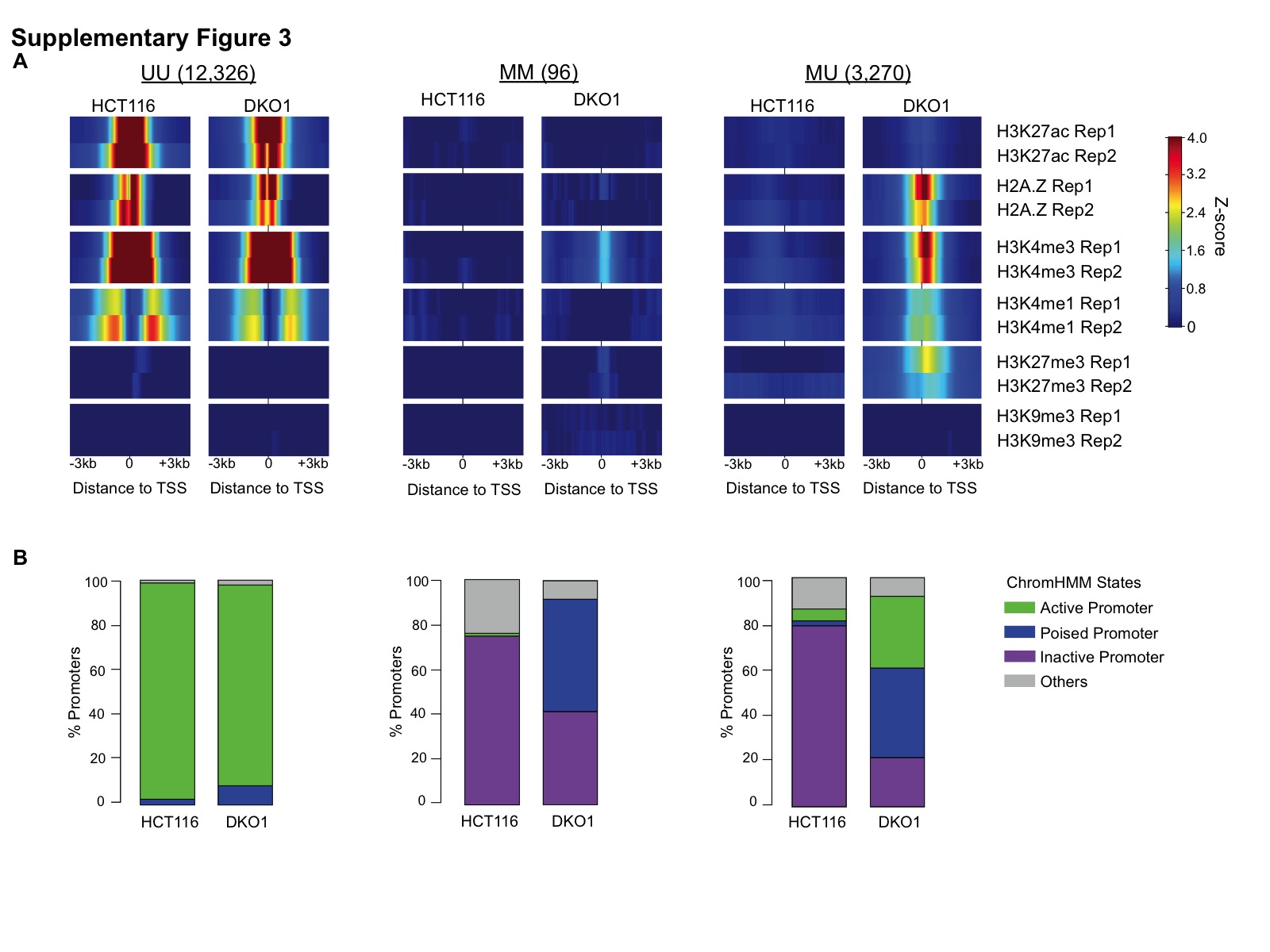

Supplement: Supplemental Material [file supp_gr.183368.114_Supp_Figure3.jpg]

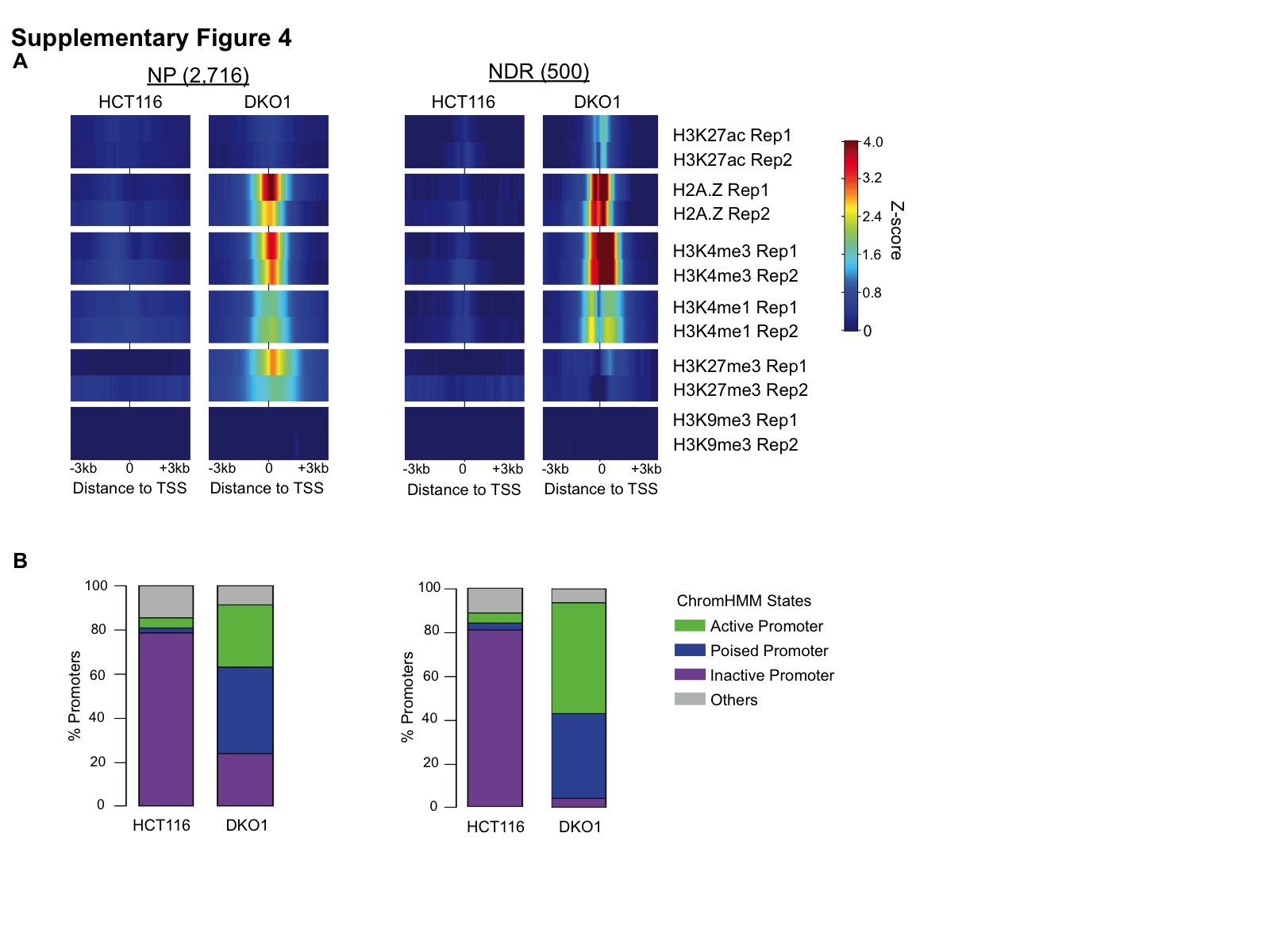

Supplement: Supplemental Material [file supp_gr.183368.114_Supp_Figure4.jpg]

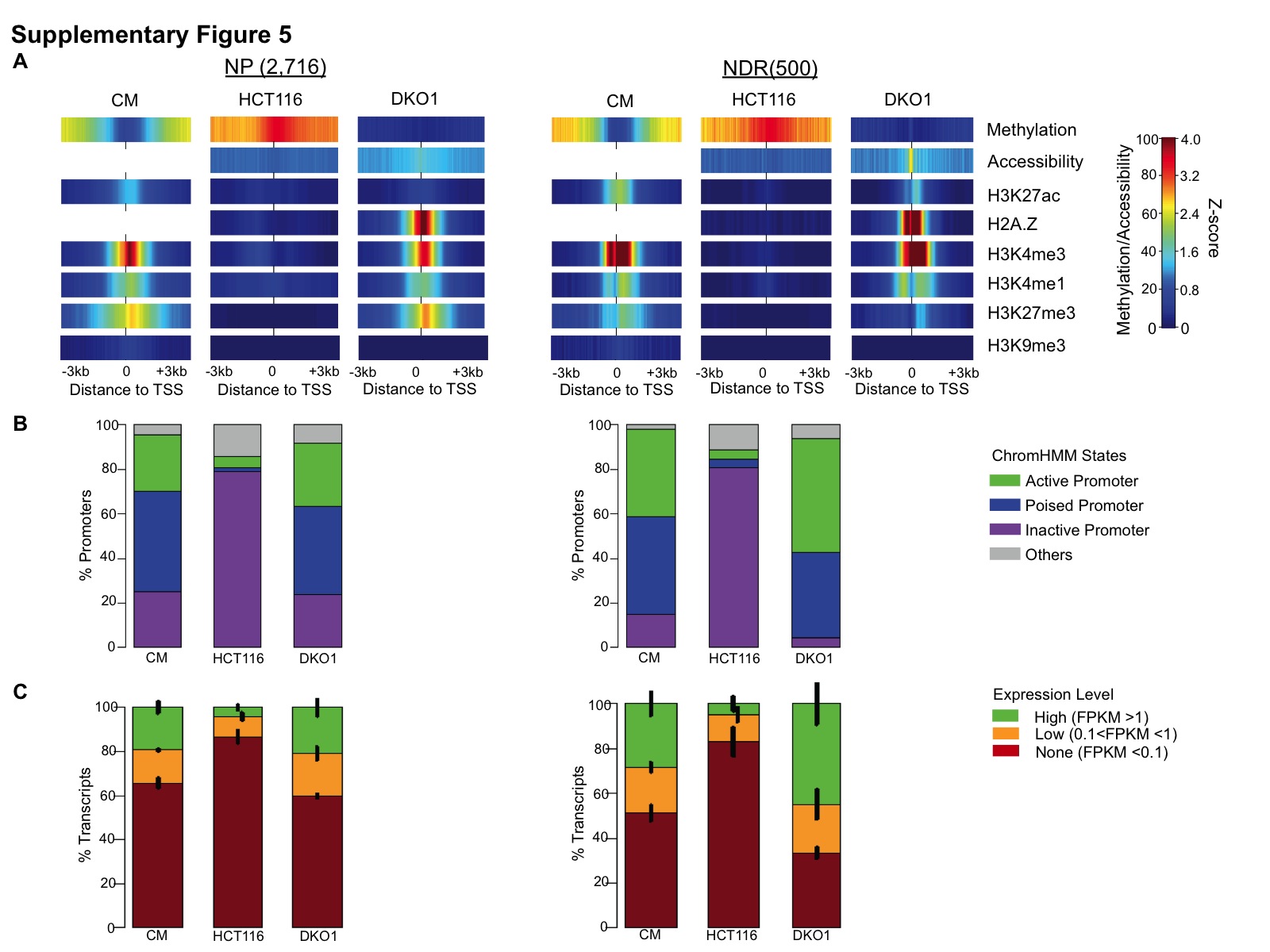

Supplement: Supplemental Material [file supp_gr.183368.114_Supp_Figure5.jpg]

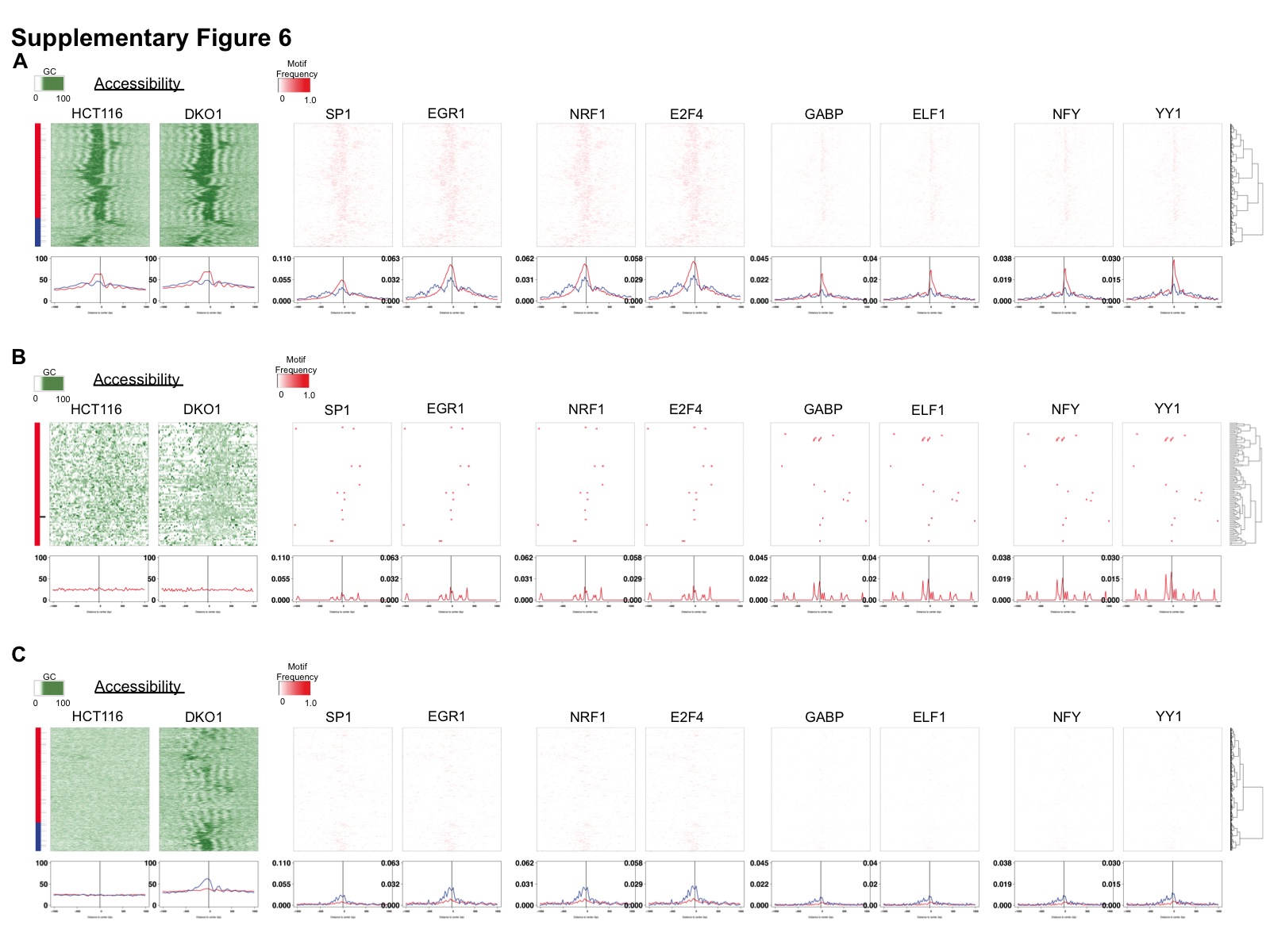

Supplement: Supplemental Material [file supp_gr.183368.114_Supp_Figure6.jpg]

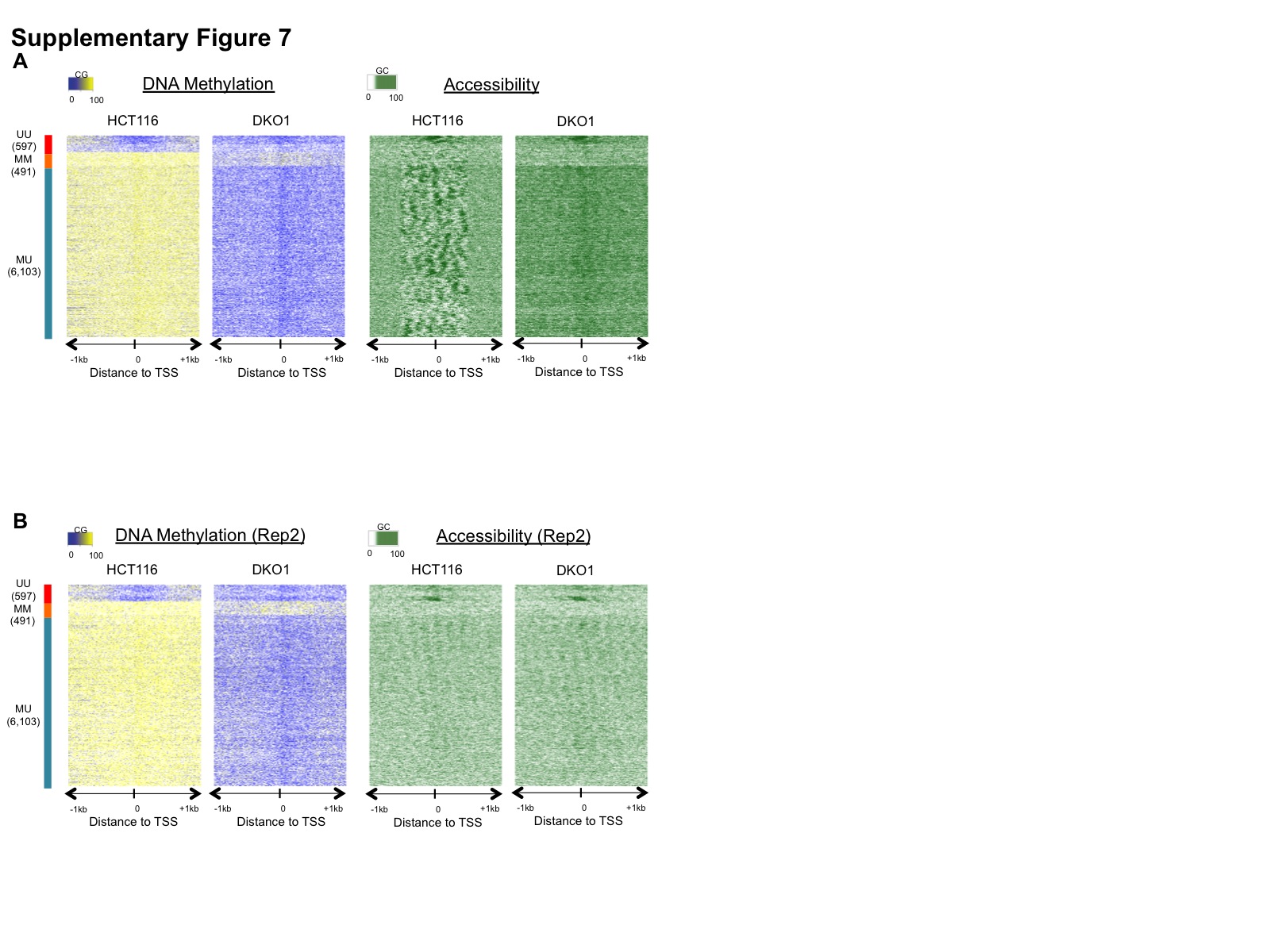

Supplement: Supplemental Material [file supp_gr.183368.114_Supp_Figure7.jpg]

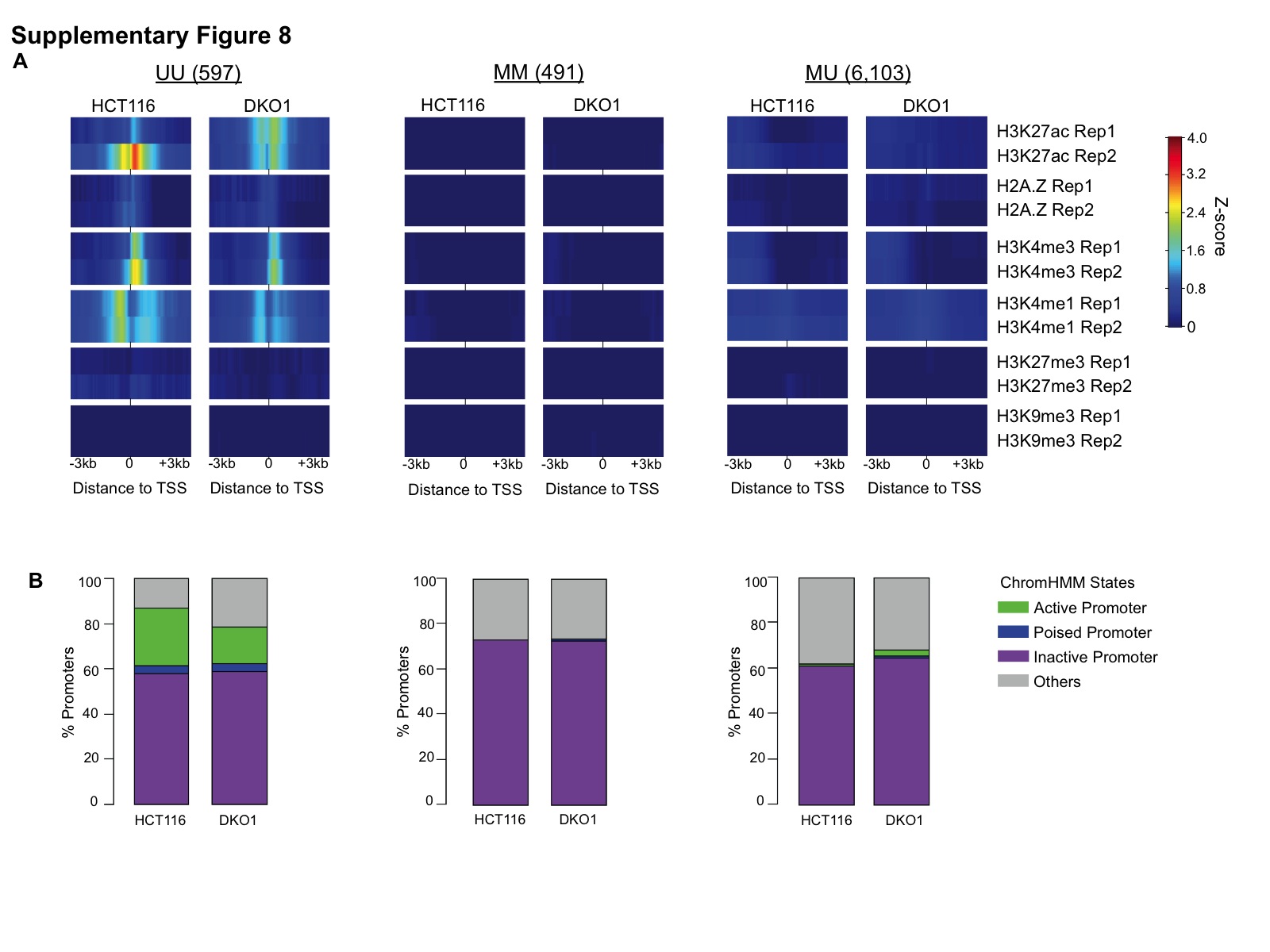

Supplement: Supplemental Material [file supp_gr.183368.114_Supp_Figure8.jpg]

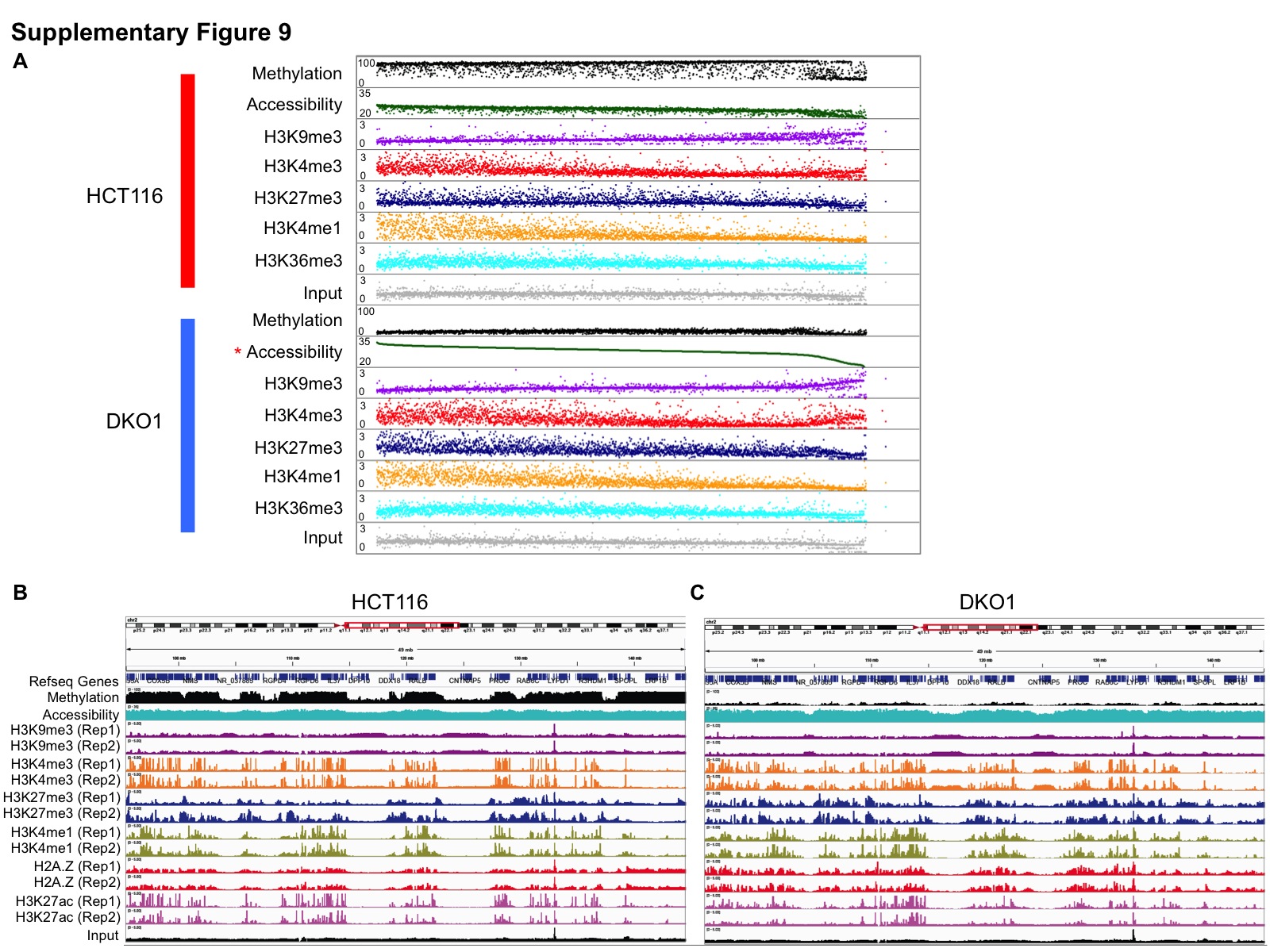

Supplement: Supplemental Material [file supp_gr.183368.114_Supp_Figure9.jpg]

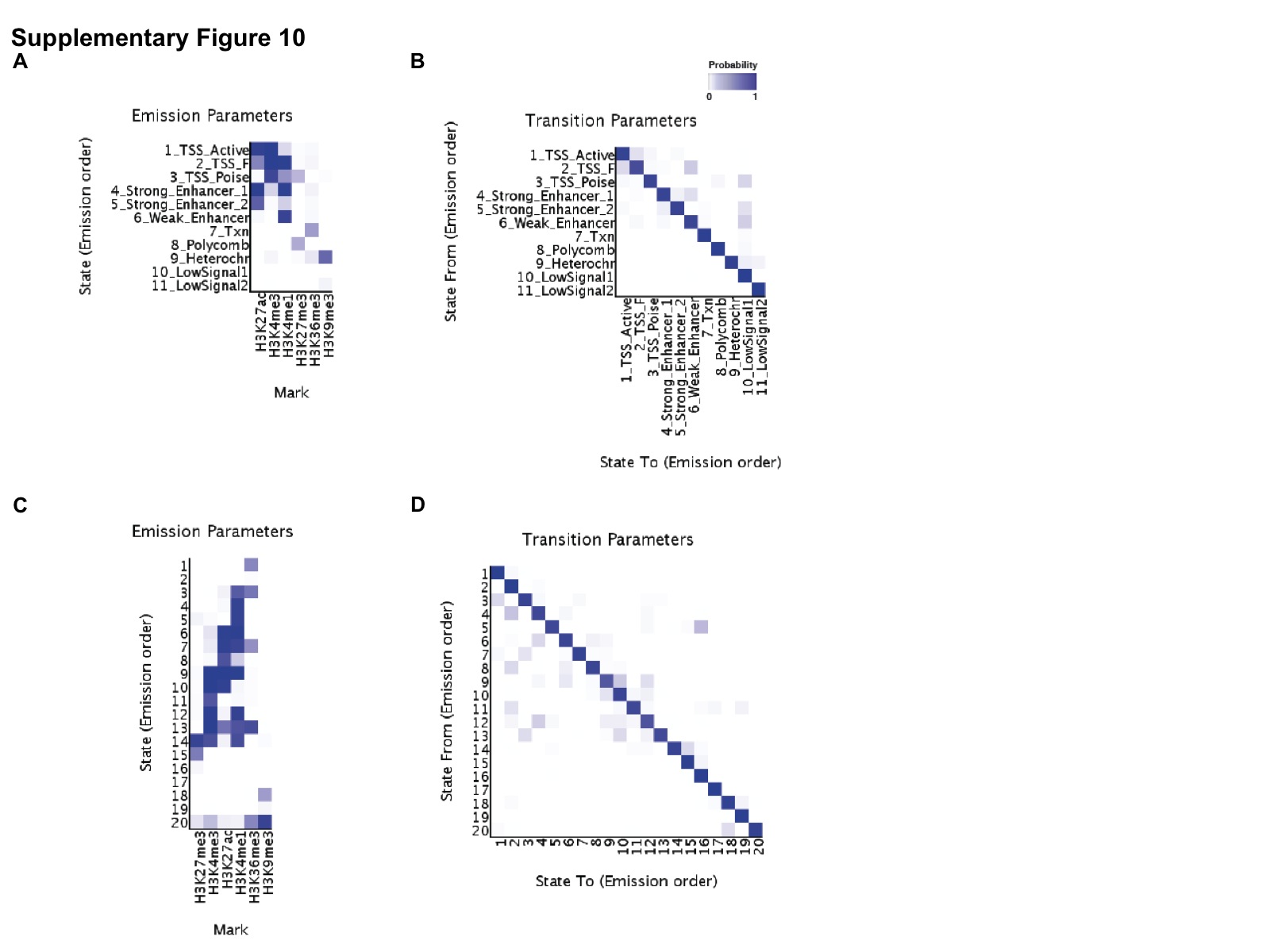

Supplement: Supplemental Material [file supp_gr.183368.114_Supp_Figure10.jpg]
