# Supplementary material for: The role of DNA methylation in directing the functional organization of the cancer epigenome
Source: Genome Res. 2015 Apr;25(4):467–77. doi: 10.1101/gr.183368.114 (PMC4381519; doi:10.1101/gr.183368.114)
Supplement: Supplemental Material [file supp_gr.183368.114_Supp_Methods.docx]

**The role of DNA methylation in directing the functional organization of the cancer epigenome**

Fides D Lay^1,2*^, Yaping Liu^2,3*^, Theresa K Kelly^1, #^, Heather Witt^1^, Peggy J Farnham^1^, Peter A Jones^1,5+^, Benjamin P Berman^3,4+^

^1^Department of Biochemistry and Molecular Biology, Norris

Comprehensive Cancer Center, Keck School of Medicine, University of Southern

California, Los Angeles, CA USA

^2^ Program in Genetic, Molecular and Cellular Biology, Keck School of Medicine, University of Southern California, Los Angeles, CA USA

^3^USC Epigenome Center, University of Southern California, Los Angeles, CA USA

^4^Department of Preventive Medicine, University of Southern California, Los Angeles, CA USA

^5^Van Andel Institute, Grand Rapids, MI USA.

Yaping Liu, present address: MIT Computer Science and Artificial Intelligence Laboratory The Broad Institute of MIT and Harvard, Cambridge, MA

Theresa K Kelly, present address: Active Motif, Carlsbad, CA.

Benjamin P Berman, present address: Bioinformatics and Computational Biology Research Center, Cedars-Sinai Medical Center, Los Angeles, CA

Correspondence should be addressed to [benjamin.berman@cshs.org](mailto:bberman@usc.edu) or [pjones@med.usc.edu](mailto:pjones@med.usc.edu)

**Supplemental Methods**

**1.DNA methylation and accessibility analysis**

1.1. NOMe-seq reads preprocessing, mapping and methylation/accessibility extraction

Fastq-mcf in ea-utils.1.1.2-484 was used to detect and trim adapter in the fastq files with parameters -k 0 -q 3. Inverted duplicate reads were filtered out when two ends of the reads were identical at bisulfite space (“T” in the 1^st^ end are considered to identical as “C” in 2^nd^ end, while “A” in the 2^nd^ end are considered to identical as “G” in 1^st^ end) in the first 20bp. After the preprocessing of reads, BSMAP v2.6 was used to map reads on hg19 reference genome with parameters -s 16 -v 10 -q 2. Multiple bam files for each biological library were merged as one bam file with the same sample ID. AddOrReplaceReadGroups.jar and MarkDuplicates.jar in Picard v1.46 were used to add read group and mark PCR duplicated reads in the bam files. Bis-SNP v0.82 wrapped in bissnp_easy_usage.pl was used to extract homozygous GCHs and HCGs methylation level with parameters --nomeseq --outMode 9 --nt 12 --mem 15 --recal –indel. The details in each step were: **1)** realign reads around known indels (1000G_phase1.indels.hg19.sort.vcf and Mills_and_1000G_gold_standard.indels.hg19.sites.sort.vcf were downloaded from 1000G project’s ftp site: <ftp://gsapubftp-anonymous:@ftp.broadinstitute.org/bundle/2.5/hg19/>). **2)** recalibrated reads base quality score. (dbsnp_135.hg19.sort.vcf was downloaded from 1000G project’s ftp site as described above); **3)** genotyping and extract GCH, HCG methylation level. dbSNP v135 was used during the genotyping. **4).** HCG or GCH that passed the following filters was used for the downstream analysis: (a) homozygous genotype with quality score more than 20, (b) strand bias less than -0.02, (c) less than 2 SNPs existed in the +/-10bp window. Bad Reads, such as PCR duplicated reads/non-unique aligned reads/bisulfite-incomplete conversion reads, were filtered as the default setting in Bis-SNP.

1.2. CGI and non-CGI TSS definition

knownGene list was downloaded from UCSC Table Browser at Aug.08.2013. TSS was extracted from each gene. Duplicated TSSs with same coordinate and strand were merged. For CpG island (CGI) promoters, we merged the Takai-Jones definition (Takai and Jones 2002) and Gardiner-Garden M, Frommer M definition (Gardiner-Garden M, Frommer M, 1987), and extended each end by 200bp. Promoters that overlapped with this definition were called as CGI promoters. For non-CGI promoters, we merged the Takai-Jones, Gardiner-Garden M, Frommer M and Irizarry CGI definition (Wu et al. 2010), extending each end by 500 bp. Promoters that did not overlap with this definition were defined as non-CGI promoters.

1.3. Global DNA methylation and accessibility changes

We used 200bp sliding window containing at least 10 GCHs with at least 2 C/T reads. Sliding on each GCH, the mean GCH methylation level was calculated as the total number of methylated C reads divided by total number of C/T reads in the window. The same procedure was applied for HCG. In Supplemental Figure 1c, only windows with enough GCHs and HCGs are kept (i.e. at least 10 GCHs+HCGs, GCH and HCG should both exist in both of HCT116 and DKO1 cells in the window. Each informative GCH/HCG should have at least 2 C/T reads covered).

**2.ChIP-seq analysis**

2.1. chromHMM

Reads in two biological replicates were pooled together, excluding non-uniquely aligned reads and PCR duplicates. ChromHMM training was performed using the default setting and with the following ChIP-seq datasets: H2A.Z, H3K4me3, H3K27ac, H3K4me1, H3K27me3, H3K36me3, H3K9me3 and Input data from HCT116, DKO1 and publically available colonic mucosa (GSM621673, GSM621672, GSM621670, GSM621671, GSM621668, GSM621669. We trained several models with the states ranging from 2 states to 20 states. We decided to use the 11 state model for the analysis since it captured all the key interactions between the chromatin marks and larger number of states did not gain more different interactions [Kasowski et al. 2013 Science]. The state emission probability matrix and transition probability matrix with 11 states and 20 states are shown in Supplemental Figure 10 respectively. Regions were decoded and labeled using the state with the maximum posterior probability. Genomic regions devoid of histone modifications are labeled as “low signal.”

2.2 Genomic enrichment of NDRs on chromHMM states categories

Promoter states were separated into CGI or non-CGI category. Weak enhancer state was separated into two states based on whether or not they overlapped CTCF motifs as defined by factorbook (Wang et al. 2013). IntersectBed in bedtools with parameters -wa -u was used to calculate the overlap between NDRs and chromHMM states. Random segments with same length distribution and same numbers as NDRs at each cluster in Supplemental Figure 2 were made for the calculation of genomic enrichment.

2.3. Long-range chromatin structure changes

Takai-Jones, Gardiner-Garden-Frommer and Irizarry definition were merged into CGI_list.bed. Custom perl script alignWigToWindow.pl was used for long-range chromatin structure changes analysis with the following parameters: --window 1000000 --not_include CGI_list.bed Reference genome were divided into 1Mb non-overlapped windows. Regions that overlapped with CGI_list.bed in each window were masked. Means of the wiggler normalized signals for ChIP-seq, WCG methylation for DNA methylation and GCH methylation for accessibility in each window were calculated and then sorted by the accessibility level in DKO1 cells.

**3. RNA-seq analysis**

TSS shared by multiple transcripts/genes was assigned to the transcript with higher mean FPKM value between two replicates of HCT116 and DKO1. Smoothscatter function in R v3.0.0 was used to visualize the replicates consistency (Supplemental Figure 11).

**4. GREAT analysis**

Each of the four NDR clusters in Figure 1a was further separated into two categories: distal (2kb away from TSS. A combined TSS category from knownTSS, GencodeBasicV17 TSS, FANTOM4 TSS) and proximal (100bp around knownTSS).

GREAT v2.02 (http://bejerano.stanford.edu/great/public/html/) was used to analyze these NDR clusters functional annotation and enrichment. Hg19 whole genome was used as background and basal plus extension option was applied for the associated genomic regions.

**5. Motif analysis**

findMotifsGenome.pl in HOMER v4.3 was used to analyze motif enrichment in the distal and proximal NDRs of each cluster in Figure 1a. The parameters *-size given -mask -mset vertebrates* were applied. Motif heatmaps in Supplemental Figure 9 were generated by using custom perl script alignWigToBed.pl to visualize the motif frequency near each CGI TSS category (motif coordinates were from Factorbook and only the motifs with frequency peak more than 0.03 at TSS were shown)

**6. External Datasets**

6.1. DNase-seq data in HCT116

The big wig files of two DNase-seq replicates data in HCT116 were downloaded from ENCODE project (http://genome.ucsc.edu/cgi-bin/hgFileSearch?db=hg19). They were visualized by custom perl script alignWigToBed.pl by the hierarchical clustering order of accessibility level in both of HCT116 and DKO1 near NDRs.

6.2. WGBS data in normal colonic mucosa

Whole genome bisulfite sequencing data was obtained from dbGaP (accession code: PHS000385).

6.3. ChIP-seq of normal colonic mucosa

ChIP-seq data of normal colonic mucosa was obtained from Roadmap Epigenomics Mapping Consortium (REMC). According to REMC’s guideline, the tissue was surgically removed from an individual with cancer of the cecum, but have normal colonic mucosa.

6.4. Other TSS definitions

GencodeBasicV17 TSS was downloaded from UCSC Table Browser. FANTOM4 TSS was downloaded from FANTOM project (http://fantom.gsc.riken.jp/4/download/).

**7. Source code availability**

Each analysis uses a custom script, which is available from Github code repository (https://github.com/dnaase/Bis-tools).

Filtering of low quality NOMe-seq reads: inv_dups_check.pl, wrap_fastq-mcf.pl

GCH/HCG methylation extraction: bissnp_easy_usage.pl

Visualization of NOMe-seq & ChIP-seq data in heatmap, density bar chart and average plot: alignWig2bed.pl

Autocorrelation analysis: AlignWig2BedMatrix2CorMatrix.java

ChIP-seq Bam to normalized Z score signal: bam2normalizedwig.pl

Beta-binomial HMM NDR detection: Bis-seg.pl

Global DNA methylation and accessibility changes detection by sliding window approach: DmrInBisulfiteVcfWalker.java

Genomic enrichment of NDRs on chromHMM states categories: annotateBed_2way.pl

Long-range chromatin structure changes: alignWig2Window.pl

**Supplemental Figure Legends**

**Supplemental Figure 1: NOMe-seq consistently detects NDRs and changes in NDRs following global hypomethylation.** A-C) Same 16,245 NDR regions shown in Fig. 1A, but for second replicates of NOMe-seq (1A), ChIP-seq (1C), and ENCODE DNase-seq (1C). D) Average levels of methylation and accessibility in HCT116 and DKO1 cells were plotted for each NDR cluster. The most dramatic change in accessibility following DNA methylation loss was observed in C3 (previously shown in Figure 1C), while C1, C2 and C4 show similar accessibility profiles between the parent and derivative cells. E) Each NDR in both cell lines was annotated based on its chromatin state as defined by chromHMM model and the distribution of chromatin states within each cluster is shown as a bar graph. The distribution is compared to randomized segments from each cell type to show the enrichment of specific chromatin state in either cell type. The total number of NDRs contained in each cluster is indicated in the parentheses. Low signal refers to genomic regions that lack histone modification signals F) Motifs that are significantly enriched in de novo NDRs in C3 are shown and p-values are given in parentheses. The analysis was done using HOMER (Supplemental Methods). G) Of all NDRs detected in K562 cells, 4072 were found within strong enhancers that were unique to the cell type. These enhancers- associated NDRs were unmethylated, accessible and flanked by well-positioned nucleosomes in K562, but were unmethylated and inaccessible in HCT116 cells. DNA methylation loss surrounding the K562-specific enhancers did not result in increased accessibility and nucleosome phasing in DKO1 cells.

**Supplemental Figure 2: Methylated CGI promoters lack accessibility and nucleosome phasing.** A) Same 15,638 CGI promoter regions shown in Fig. 2A, but clustered based on accessibility in HCT116 rather than DKO1. B) Zoom of the MM (top panel) and MU (bottom panel) classes shown in (A). C) NOMe-seq reads for the second biological replicate of HCT116 and DKO1 cells were aligned to +1/-1kb of the TSS and heatmaps were generated in the same order as Figure 2A to show the reproducibility of NOMe-seq analyses. D) Zoom of MM (top panel) and MU (bottom panel) classes shown in (C).

**Supplemental Figure 3: Chromatin remodeling occurs in CGI promoters in the absence of DNA methylation.** A) Enrichment level for two biological replicates of each histone mark, expressed in terms of z-score is shown +/-3kb around the TSS as the average of all promoters in each class. Z-score was calculated based on normalized experimental wiggler value of each histone mark compared to the input. The number of promoters in each cluster is indicated in the parentheses. Rep1 is previously shown in Figure 2C. B) Distribution of chromHMM states in each CGI promoter class is shown as a bar chart.

**Supplemental Figure 4: MU promoters gain active and poised chromatin states in DKO1 cells.** A) Enrichment level for two biological replicates of each histone mark is shown for MU promoters that gain nucleosome phasing (NP) and accessibility (NDR). The number of promoters in each cluster is indicated in the parentheses. Rep1 is previously shown in Figure 3b. B) Distribution of chromHMM states in each subclass of MU promoters is shown as a barchart.

**Supplemental Figure 5: DKO1 cells gain a normal-like chromatin landscape at MU CGI promoters.** A) Average DNA methylation, accessibility and histone mark enrichment levels are shown +/-3kb around the TSS as the average of all promoters in each class for colonic mucosa (CM), HCT116 and DKO1 cells at MU promoters that gain nucleosome phasing (NP) and accessibility (NDR). DNA methylation and accessibility levels are shown as percentage whereas histone enrichment levels are shown as z-scores. B) Distribution of chromatin states and C) expression levels for each promoter class in all cell type is shown as a bar chart.

**Supplemental Figure 6: Unmethylated and accessible CGI TSSs are enriched for general transcription factor motifs.** A) Frequencies of transcription factor motifs near UU, B) MM and C) MU promoters in CGIs were calculated using HOMER and motifs with frequency peak > 0.03 are shown. Coordinate of the motifs were obtained from Factorbook.

**Supplemental Figure 7: Weakly-phased nucleosome pattern is consistently detected in methylated non-CGI promoters.** A) NOMe-seq reads were aligned to 7,191 annotated non-CGI TSS and promoters were categorized based on the methylation levels in both cell types as followed: Unmethylated in HCT116 and Unmethylated in DKO1 (UU), Methylated in HCT116 and Methylated in DKO1 (MM) and Methylated in HCT116 and Unmethylated in DKO1 (MU). The number of promoters that fall in each class is shown on the left. Heatmaps were generated for DNA methylation and accessibility +/-1kb from the TSS and reverse hierarchical clustering of each row was done based on the accessibility pattern of HCT116 cells for each promoter group. B) NOMe-seq reads for the second biological replicate of HCT116 and DKO1 cells were aligned to +1/-1kb of the TSS and heatmaps were generated in the same order as Figure 5a to show the reproducibility of NOMe-seq analyses.

**Supplemental Figure 8: The majority of non-CGI promoters adopt a closed chromatin configuration.** A) Enrichment level for two biological replicates of each histone mark, expressed in terms of z-score is shown +/-3kb around the TSS as the average of all promoters in each class. Z-score was calculated based on normalized experimental wiggler value of each histone mark compared to the input. The number of promoters in each cluster is indicated in the parentheses. Rep1 is previously shown in Figure 4C. B) Distribution of chromatin states for each promoter class in all cell type is shown as a bar chart.

**Supplemental Figure 9: Long range decrease in accessibility is associated with the increase of H3K9me3 blocks in PMDs.** A) Ranked dot plot is shown to describe long-range chromatin changes. Each dot or data point represents the average levels of methylation, accessibility and z-scores (y-axes) for a 1Mb genomic window which exclude CGI (n=3,089). The data points are plotted and ordered based on the accessibility level of DKO1 cells. B) IGV browser shot of a 49Mb genomic window located in chromosome 2 of HCT116 and B) DKO1 cells showing the levels of methylation and accessibility as well as enrichments of histone marks in biological replicates.

**Supplemental Figure 10: Determination of functional chromatin states using the chromHMM model.** ChIP-seq data were trained to generate A-B) 11 and C-D) 20 chromatin states models based on the listed histone modifications. A&C) Emission and B&D) transition parameters for each model are shown.
