# Supplementary material for: The role of DNA methylation in directing the functional organization of the cancer epigenome
Source: Genome Res. 2015 Apr;25(4):467–77. doi: 10.1101/gr.183368.114 (PMC4381519; doi:10.1101/gr.183368.114)
Supplement: Supplemental Material [file supp_gr.183368.114_Supp_Table1.docx]

**Supplementary Table 1a: NOMe-seq/WGBS Libraries Information**

| **Sample Name** | **Sample Description** | **Library** | **GEO/dbGap ID** | **Source** | **Total Mapped Reads** | **Informative Reads** | **Incomplete Bisulfite Conversion Rate (TCC in chrM)** |
| --- | --- | --- | --- | --- | --- | --- | --- |
| KEL656A167 | HCT116(rep1) | NOMe-seq | GSM1420150 | internal | 650,972,087 | 448,082,341 | 0.82 |
| KEL656A258 | HCT116(rep2) | NOMe-seq | GSM1416976 | internal | 332,235,767 | 278,710,308 | 0.22 |
| KEL656A168 | DKO1(rep1) | NOMe-seq | GSM1420151 | internal | 408,547,447 | 313,683,520 | 1.48 |
| KEL656A259 | DKO1(rep2) | NOMe-seq | GSM1416977 | internal | 343,196,628 | 299,268,908 | 0.23 |
| BENATURE_1_normal | Colonic Mucosa | WGBS | PHS000385 | public (Berman et al. 2012) | 1,381,307,342 |  | 0.70 |
| KEL656A310 | K562 | NOMe-seq | GSM1583563 | internal | 757,776,380 |  | 0.9 |

**Supplementary Table 1b: ChIP-seq Libraries Information**

| **Sample Name** | **Sample Description** | **Library** | **GEO/SRA/**  **ENCODE ID** | **Source** | **Total Mapped Reads** | **Informative Reads** |
| --- | --- | --- | --- | --- | --- | --- |
| KEL656A202 | HCT116(rep1) | H3K27ac | GSM1415876 | internal | 5,510,801 | 4,581,683 |
| wgEncodeSydhHistoneHct116H3k27acUcdAlnRep1 | HCT116(rep2) | H3K27ac | GSM945853 | public (ENCODE) | 13,523,797 | 12,311,578 |
| KEL656A217 | HCT116(rep1) | H2A.Z | GSM1420152 | internal | 10,722,196 | 10,077,735 |
| KEL656A246 | HCT116(rep2) | H2A.Z | GSM1415873 | internal | 30,037,296 | 22,325,014 |
| KEL656A218 | HCT116(rep1) | H3K4me3 | GSM1415874 | internal | 13,680,763 | 11,804,541 |
| KEL656A289 | HCT116(rep2) | H3K4me3 | GSM1420153 | internal | 38,685,158 | 32,559,382 |
| KEL656A214 | HCT116(rep1) | H3K4me1 | GSM1415875 | internal | 10,744,842 | 8,866,167 |
| KEL656A293 | HCT116(rep2) | H3K4me1 | GSM1420154 | internal | 41,504,399 | 35,291,365 |
| FC64LGK_L6 | HCT116(rep1) | H3K27me3 | GSM1420155 | internal | 37,427,240 | 31,370,059 |
| KEL656A215 | HCT116(rep2) | H3K27me3 | GSM1415877 | internal | 21,780,932 | 20,629,078 |
| ENCBS471AAA | HCT116(rep1) | H3K36me3 | ENCFF002AAN | public (ENCODE) | 22,448,528 | 18,718,557 |
| ENCBS472AAA | HCT116(rep2) | H3K36me3 | ENCFF002AAO | public (ENCODE) | 38,923,969 | 32,347,994 |
| ENCLB555ABS | HCT116(rep1) | H3K9me3 | ENCFF002AAK | public (ENCODE) | 24,464,968 | 19,683,515 |
| ENCLB555ABT | HCT116(rep2) | H3K9me3 | ENCFF002AAM | public (ENCODE) | 36,840,118 | 29,016,007 |
| KEL656A247 | HCT116(rep1) | Input | GSM1415880 | internal | 33,738,143 | 21,150,576 |
| wgEncodeSydhHistoneHct116InputUcdAlnRep1 | HCT116(rep2) | Input | GSM945855 | public (ENCODE) | 31,166,160 | 29,736,440 |
| KEL656A222 | DKO1(rep1) | H3K27ac | GSM1415884 | internal | 24,247,812 | 4,322,276 |
| FC64LKM_L6 | DKO1(rep2) | H3K27ac | GSM1420161 | internal | 26,785,591 | 22,229,197 |
| KEL656A210 | DKO1(rep2) | H2A.Z | GSM1420158 | internal | 10,486,116 | 9,799,007 |
| KEL656A248 | DKO1(rep1) | H2A.Z | GSM1415881 | internal | 35,754,339 | 26,384,495 |
| KEL656A224 | DKO1(rep1) | H3K4me3 | GSM1415882 | internal | 41,744,213 | 17,217,419 |
| KEL656A290 | DKO1(rep2) | H3K4me3 | GSM1420159 | internal | 34,699,882 | 31,117,882 |
| KEL656A223 | DKO1(rep1) | H3K4me1 | GSM1415883 | internal | 31,592,319 | 10,103,369 |
| KEL656A295 | DKO1(rep2) | H3K4me1 | GSM1420160 | internal | 46,694,645 | 29,872,359 |
| KEL656A212 | DKO1(rep1) | H3K27me3 | GSM1415885 | internal | 15,177,910 | 14,462,379 |
| KEL656A296 | DKO1(rep2) | H3K27me3 | GSM1420162 | internal | 43,893,543 | 36,031,159 |
| FC64LBK_L7 | DKO1 | H3K36me3 | GSM1415887 | internal | 34,095,023 | 27,241,487 |
| FC64LKL_L8 | DKO1(rep1) | H3K9me3 | GSM1415886 | internal | 33,252,190 | 26,572,053 |
| FC64Y36_L3 | DKO1(rep2) | H3K9me3 | GSM1420163 | internal | 14,676,889 | 11,854,251 |
| KEL656A249 | DKO1(rep1) | Input | GSM1415888 | internal | 35,265,660 | 24,379,748 |
| FC64D8G_L1 | DKO1(rep2) | Input | GSM1420164 | internal | 35,179,865 | 29,071,804 |
| GSMunknown_BI_Colonic_Mucosa_H3K27ac_32 | Colonic Mucosa | H3K27ac | NA | public (Zhu et al. 2013) | NA | 16,182,289 |
| GSM621671 | Colonic Mucosa | H3K4me3 | GSM621671 | public (Zhu et al. 2013) | NA | 12,083,710 |
| GSM621670 | Colonic Mucosa | H3K4me1 | GSM621670 | public (Zhu et al. 2013) | NA | 12,223,350 |
| GSM621673 | Colonic Mucosa | H3K27me3 | GSM621673 | public (Zhu et al. 2013) | NA | 12,199,603 |
| GSM621672 | Colonic Mucosa | H3K36me3 | GSM621672 | public (Zhu et al. 2013) | NA | 13,776,799 |
| GSM621668 | Colonic Mucosa | H3K9me3 | GSM621668 | public (Zhu et al. 2013) | NA | 11,764,307 |
| GSM621669 | Colonic Mucosa | Input | GSM621669 | public (Zhu et al. 2013) | NA | 17,863,190 |

**Supplementary Table 1c: RNA-seq Libraries Information**

| **Sample Name** | **Sample Description** | **Library** | **GEO/TCGA ID** | **Source** | **Total Mapped Reads** | **Informative Reads** |
| --- | --- | --- | --- | --- | --- | --- |
| KEL656A236 | HCT116(rep1) | RNA-seq | GSM1266733 | internal | 122,996,615 | 75,259,192 |
| WIT1251A69 | HCT116(rep2) | RNA-seq | GSM1266734 | internal | 132,341,247 | 78,317,272 |
| KEL656A237 | DKO1(rep1) | RNA-seq | GSM1266733 | internal | 113,534,563 | 69,717,449 |
| WIT1251A70 | DKO1(rep2) | RNA-seq | GSM1266734 | internal | 136,889,294 | 78,498,618 |
| TCGA-A6-5665-11A-01R-1653-07 | Colonic Mucosa | RNA-seq | TCGA-A6-5665-11A-01R-1653-07 | TCGA | 138,806,024 | 73,076,650 |
| TCGA-A6-5667-11A-01R-1723-07 | Colonic Mucosa | RNA-seq | TCGA-A6-5667-11A-01R-1723-07 | TCGA | 151,955,959 | 85,262,211 |
| TCGA-AA-3496-11A-01R-1839-07 | Colonic Mucosa | RNA-seq | TCGA-AA-3496-11A-01R-1839-07 | TCGA | 95,183,470 | 54,880,882 |
| TCGA-AZ-6605-11A-01R-1839-07 | Colonic Mucosa | RNA-seq | TCGA-AZ-6605-11A-01R-1839-07 | TCGA | 141,594,718 | 74,431,334 |
